# Switching carbon metabolic flux for enhancing the production of sesquiterpene-based high-density biofuel precursor in *Saccharomyces cerevisiae*

**DOI:** 10.1186/s13068-023-02370-8

**Published:** 2023-08-04

**Authors:** Bo Liang, Qun Yang, Xinping Zhang, Yukun Zhao, Yunhui Liu, Jianming Yang, Zhaobao Wang

**Affiliations:** 1 Energy-rich Compounds Production by Photosynthetic Carbon Fixation Research Center, Qingdao Agricultural University, Qingdao, China; 2 Shandong Key Lab of Applied Mycology, College of Life Sciences, Qingdao Agricultural University, Qingdao, China; 3 Pony Testing International Group, Qingdao, China

**Keywords:** β-Caryophyllene, MAAC pathway, Central carbon metabolic flux, *Saccharomyces cerevisiae*

## Abstract

**Background:**

Sesquiterpenes are designated as a large class of plant-derived natural active compounds, which have wide applications in industries of energy, food, cosmetics, medicine and agriculture. Neither plant extraction nor chemical synthesis can meet the massive market demands and sustainable development goals. Biosynthesis in microbial cell factories represents an eco-friendly and high-efficient way. Among several microorganisms, *Saccharomyces cerevisiae* exhibited the potential as a chassis for bioproduction of various sesquiterpenes due to its native mevalonate pathway. However, its inefficient nature limits biosynthesis of diverse sesquiterpenes at industrial grade.

**Results:**

Herein, we exploited an artificial synthetic malonic acid-acetoacetyl-CoA (MAAC) metabolic pathway to switch central carbon metabolic flux for stable and efficient biosynthesis of sesquiterpene-based high-density biofuel precursor in *S. cerevisiae*. Through investigations at transcription and metabolism levels, we revealed that strains with rewired central metabolism can devote more sugars to β-caryophyllene production. By optimizing the MVA pathway, the yield of β-caryophyllene from YQ-4 was 25.8 mg/L, which was 3 times higher than that of the initial strain YQ-1. Strain YQ-7 was obtained by introducing malonic acid metabolic pathway. Combing the optimized flask fermentation process, the target production boosted by about 13-fold, to 328 mg/L compared to that in the strain YQ-4 without malonic acid metabolic pathway.

**Conclusion:**

This designed MAAC pathway for sesquiterpene-based high-density biofuel precursor synthesis can provide an impressive cornerstone for achieving a sustainable production of renewable fuels.

**Supplementary Information:**

The online version contains supplementary material available at 10.1186/s13068-023-02370-8.

## Introduction

For decades, the excessive depletion of fossil fuels such as coal and petroleum caused atmospheric contamination, global warming and other serious issues, which impelling imperative efforts on exploiting renewable and sustainable alternatives to fossil fuels [1]. As the natural derivatives of isoprene, a variety of terpenoids have exhibited broad applications in industries of energy, food, cosmetics, medicine and agriculture [2]. Especially, several sesquiterpenes (C15) with the characteristics of compact structures and low hydrophilicity have been recognized as ideal renewable fuels, providing the attractive candidates for petroleum-independent energy [3]. For example, sesquiterpene β-caryophyllene has great potential to be used as the next-generation aircraft fuel component apart from its anti-inflammatory and antioxidant activities [4, 5]. Although sesquiterpenes are the complex secondary metabolites of plants, the production mode by extracting from their natural sources often suffers from tedious procedures, extremely low yields and restricted source of materials [6]. As for chemical approach, resource limitations and toxicity of chemical raw materials do not meet the sustainable development goals [7]. Therefore, the development of alternative green approaches for the large-scale sustainable production of sesquiterpene have drawn global attentions.

In recent years, microbial cell factories for sesquiterpene biosynthesis were regarded as an alternative to above methods due to their environmentally friendly and sustainable properties [8]. Until now, various synthetic pathways of sesquiterpene have been well-established in *Saccharomyces cerevisiae* [9]. In addition to efforts on rate-limiting enzymes engineering [10] and down-stream sesquiterpenes pathway engineering [10], strategies for enhancing acetyl-CoA supply have been also developed to meet the requirement for industrial production, including introducing heterologous routes related to acetyl-CoA synthesis [11] and compartmentalization engineering [12].

Malonic acid, or propanedioic acid, is a dicarboxylic acid with three carbons. It is well known as a competitive inhibitor of succinate dehydrogenase of TCA cycle in almost all organisms [13]. Nevertheless, it has been proved that malonic acid assimilation plays an essential role in symbiotic nitrogen metabolism [13]. Importantly, beside manufacturing applications, such as chemical synthesis of flavors, fragrances, and pharmaceuticals [14], malonic acid has also been used as a building block chemical to produce diverse high-value malonyl-CoA-derived compounds in microbial cell factories, such as fatty acids, polyketides [15] flavonoids [16] and even 3-hydroxypropionate (3-HP) [17] and glutaric acid [18]. In these pioneer works, malonyl-CoA can be generated from malonic acid through only two steps in *E. coli*, involving malonic acid transport protein and malonyl-CoA synthase, which is the shortest route relative to glucose metabolic pathway [15–18]. However, the employment of malonic acid for biosynthesis have been realized in *E. coli* rather than *S. cerevisiae* [15–18]*.* As the model eukaryotic microorganism, the introduction of malonic acid assimilation in *S. cerevisiae* microbial cell factory has great application prospects in the field of bioproduction [19].

**Fig. 1:**
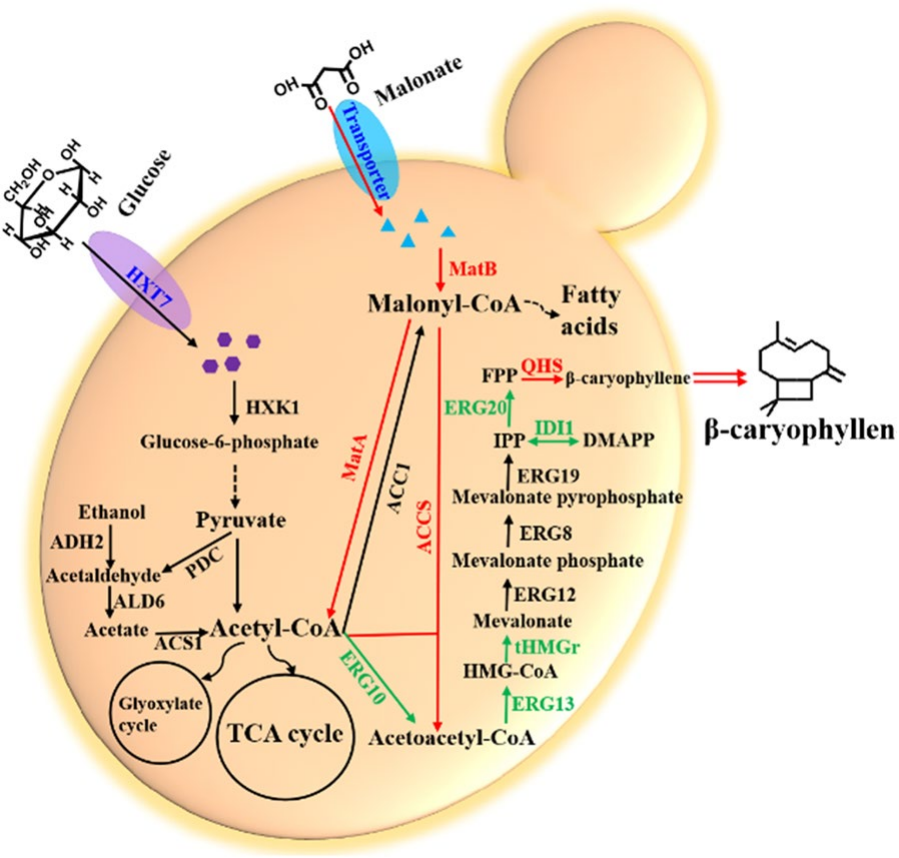
Switching carbon metabolic flux for sesquiterpenes biosynthesis in *S. cerevisiae*. Native mentalism network is depicted with dark. Malonate can be transformed into acetoacetyl-CoA via synthetic MAAC pathway (red arrows). The engineered cytosolic MVA routes (green arrows) is responsible for β-caryophyllene formation. HXT7, high-affinity glucose transporter; HXK1, hexokinase; PDC, pyruvate decarboxylase; ADH2, ethanol dehydrogenase; ALD6, acetaldehyde dehydrogenase; ACS1, acetyl coenzyme A synthetase; ACC1, acetyl Coenzyme A carboxylase; Mae I, malonate transporter; MatB, malonyl-CoA synthase; Mat A, malonyl-CoA decarboxylase; ACCS, acetoacetyl-CoA synthase; ERG10, acetyl-CoA C-acetyltransferase; ERG13, 3-hydroxy-3-methylglutaryl-CoA (HMG-CoA) synthase; tHMGr, truncated HMG-CoA reductase; ERG12, mevalonate kinase; ERG8, phosphomevalonate kinase; ERG19, mevalonate pyrophosphate decarboxylase; IDI1, isopentenyl diphosphate isomerase; ERG20, farnesyl diphosphate synthase; QHS, β-caryophyllene synthase

Although remarkable achievements have been made in the biosynthesis of sesquiterpene in *S. cerevisiae* due to its original MVA pathways, carbon and energy metabolism are strictly regulated in cells, which greatly limit the highly efficient production of sesquiterpene [20]. To overcome this issue, here we firstly developed an alternative pathway termed malonic acid-acetoacetyl-CoA (MAAC) pathway that enabling to circumvent intrinsic inefficiencies of native central metabolism and thus enhancing the productivity of the targets in *S. cerevisiae*. Until now, few groups have attempted to produce β-caryophyllene in yeast [21, 22]. However, the titers failed to meet demands of industrial production [21, 22]. In our present work, to maintain stable yield of β-caryophyllene in *S. cerevisiae*, the genome sequence has been successfully reconstructed by introducing endogenous and heterogeneous genes. Carbon isotope labeling experiment and transcription analysis revealed that the rewired central carbon metabolic network efficiently promoted mevalonate pathway (MVA pathway) by increasing the availability of central metabolic intermediates acetyl-CoA and acetoacetyl-CoA (Fig. 1). Finally, β-caryophyllene production was significantly boosted after optimization of fermentation conditions, and these results further confirmed our proposed mechanisms to increase production mediated by MAAC pathway. Moreover, the recruitment of this route in biosynthesis of another sesquiterpene (C15) β-elemene also led to enhanced production and the titer of 984.36 ± 50.31 mg/L reached to the highest level ever reported. Therefore, this study expands the application fields of malonic acid assimilation pathway, from biosynthesis of malonyl-CoA-derived valuable compounds to biosynthesis of acetyl-CoA-derived natural high-value products.

## Results

### Establishing the biosynthesis of β-caryophyllene in *S. cerevisiae* through Cas9-based genome editing

We designed a biosynthetic pathway for β-caryophyllene production from the cytosolic acetyl-CoA in yeast through endogenous MVA pathway and heterologous β-caryophyllene synthases (QHS) (Fig. 1). QHS has an ability of converting farnesyl pyrophosphate (FPP) to β-caryophyllene [23]. As a starting point, we used a yeast platform strain (CEN.PK2-1D) as chassis cells. Genome has been reconstructed by Cas9-based genome editing method.

**Fig. 2:**
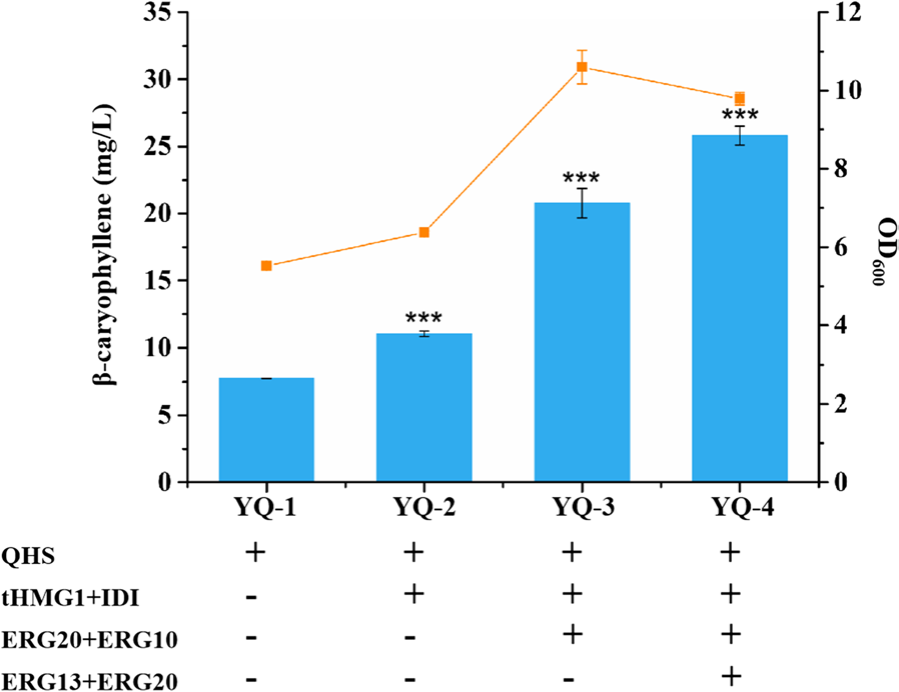
Metabolic engineering of MVA pathway for stable biosynthesis of β-caryophyllene. The concentrations of product were determined after 72 h of culture in YPD medium at 30 °C. Error bars represent the standard deviations of three replicates. *** symbolizes significant pairwise differences with YQ-1, *p* < 0.001

To develop a genetically stable and efficient strain that could overproduce β-caryophyllene, QHS encoding genes from *A. annua* was integrated into genome loci of CEN.PK2-1D, due to the higher titer of β-caryophyllene reported in early studies [24]. The resulting strain was named strain YQ-1 with a production of β-caryophyllene of 7.75 ± 0.01 mg/L (Fig. 2). At this point, we obtained an engineered yeast, which could produce β-caryophyllene in a stable manner.

To further improve the titer of β-caryophyllene, we focused on MVA pathway engineering in subsequent work. Overexpression of truncated HMG-CoA reductase isozyme 1 (tHMG1), lacking a regulatory domain, is currently the most effective strategy for sesquiterpenes overproduction in *S. cerevisiae*. In addition to tHMG1, other key enzymes were also as targets for the metabolic engineering of sesquiterpenes production in yeast, including IDI1, ERG20, ERG13, and ERG10 [10, 20]. Hence, we integrated an additional of tHMG1, IDI1, ERG20, ERG13, and ERG10 into the genome of YQ-1 to generate strain YQ-2, YQ-3 and YQ-4. As expected, the resulted strains lifted the titer of target by nearly 1.42, 2.68 and 3.33 folds, respectively (Fig. 2).

### The introduction of the MAAC pathway to enhance the production of sesquiterpenes

Previous studies have been reported that acetyl-CoA was the crucial precursor for sesquiterpenes production [20]. Enhancements of acetyl-CoA supply for overproduction of sesquiterpenes have been accomplished by reconstructing metabolic pathways or tailoring cell organelles into microbial cell factories in previous studies [11, 12]. Nevertheless, achieving economically viable titers is still a major roadblock in the process of reaching industrial production of high-value compounds. Aside from acetyl-CoA, acetoacetyl-CoA is a next vital precursor in MVA pathway, which can be obtained by the action of ERG10 from two molecules of acetyl-CoA. Thus, the increased supply of acetoacetyl-CoA was an alternative way to stimulate MVA pathway. A recently identified acetoacetyl-CoA synthase (ACCS) from Streptomyces sp. strain CL190, which catalyzes the condensation of malonyl-CoA and acetyl-CoA to generate acetoacetyl-CoA, has been proposed as a potential target to enhance production of acetoacetyl-CoA derived compounds. Unfortunately, production of farnesene did not benefit from ACCS expression [25]. It was speculated that malonyl-CoA represents a critical precursor for this pathway as well as for fatty acid synthesis and, therefore, its availability for the mevalonate pathway might be insufficient. Nevertheless, the blockage of fatty acid formation led to severe damage of cell growth [25]. Hence, we speculated the introduction of heterogeneous malonyl-CoA producing route might be a promising solution to balance cells growth and the biosynthesis of β-caryophyllene. In the present work, malonic acid assimilation pathway accompanied by ACCS or malonyl-CoA decarboxylase (MatA) were recruited to rewrite the central metabolism in two ways: (i) directly increasing supply of acetoacetyl-CoA or acetyl-CoA from non-fermentable carbon source malonic acid; (ii) reducing the carbon flow from acetyl-CoA to fatty acid biosynthesis by increasing the source of malonyl-CoA. Thus, the obvious reinforcement of sesquiterpenes production in current study was benefited from providing abundant malonyl-CoA for biosynthesis of β-caryophyllene and fatty acids. Shake-flask tests were carried out to determine the effects of the synthetic MAAC pathway in sesquiterpenes bioproduction.

**Fig. 3:**
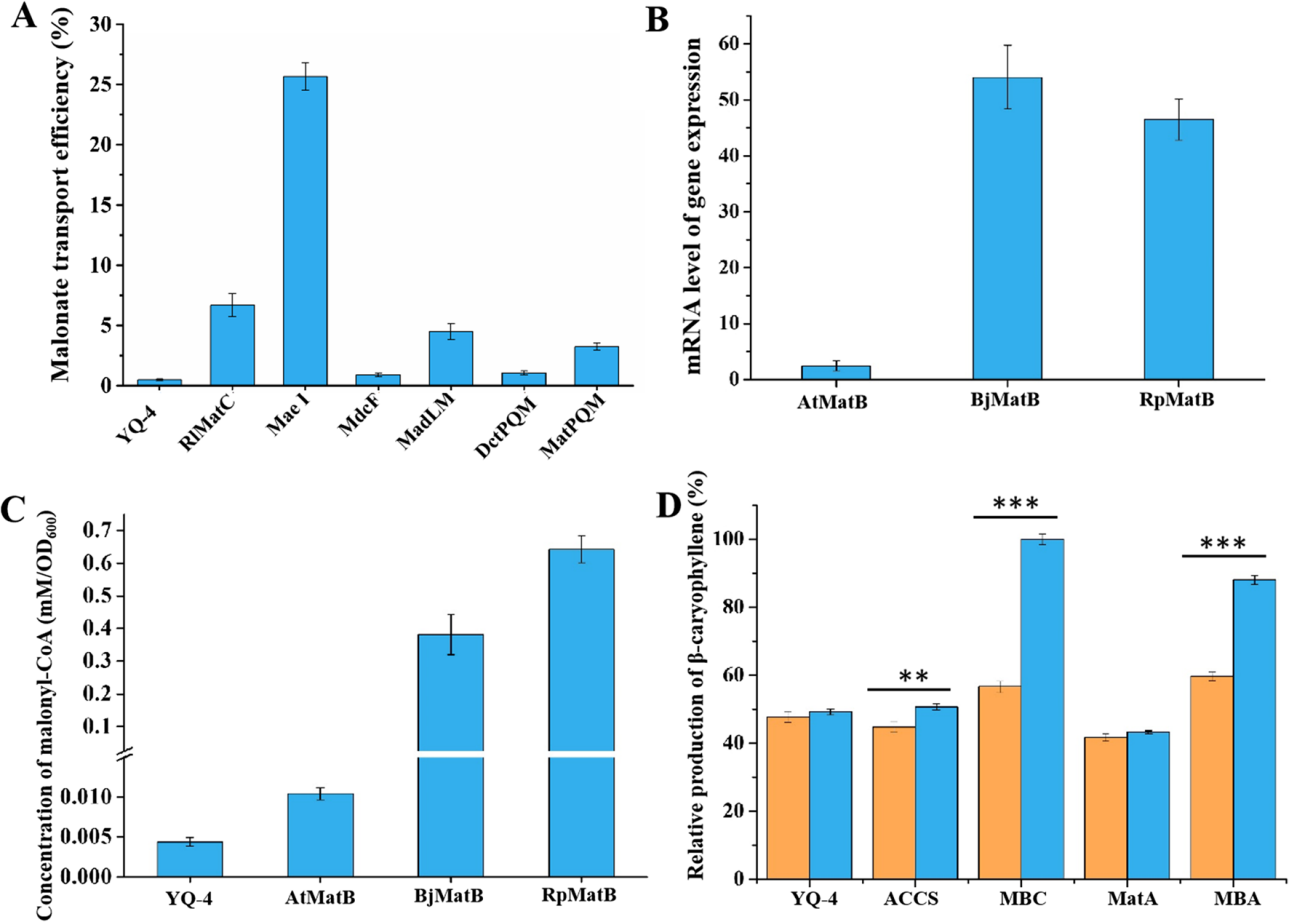
The construction of synthetic MAAC pathway for enhancing the production of β-caryophyllene. **A** Malonate transport efficiency by the action of malonic acid transport protein originated from various microorganisms. Recombinant strains were cultured in the YPD medium supplemented with 60 mM of malonate for 12 h. Then samples were taken for detection of malonic acid. **B** Gene expression levels of MatB from different origins in strains. Relative expression level was the intensity of MatB divided by the intensity of internal reference ALG9. **C** The amounts of malonyl-CoA in cells expressing Mae I and different sources of MatB after 36 h cultivation. **D** Relative β-caryophyllene production via four metabolic routes with malonate (blue bar) or not (yellow bar). The production of β-caryophyllene through MBC pathway in the presence of malonate was regarded as 100%. Data are presented here as mean values ± standard deviation (SD) calculated from n = 3 biological replicates. ** indicates significant pairwise differences between two bars, *p* < 0.01, *** indicates significant pairwise differences between two bars, *p* < 0.001

First of all, membrane transport limitations were addressed by incorporation of malonate transporters due to the toxicity of malonate to cells [26]. Until now, several transporters responsible for malonate transportation have been identified [17]. To achieve the superior activity in *S. cerevisiae*, we screened seven malonate transporters with reported activity on malonate transportation via expression from a plasmid pRS41H in CEN.PK2-1D, including *Rl*MatC from *Rhizobium leguminosarum bv trifolii* [13], Mae I from *Schizosaccharomyces pombe* [27], MdcF from *Klebsiella pneumoniae* [28], MadLM from *Acinetobacter baylyi* [29], tripartite ATP-independent periplasmic (TRAP) transporters from *Rhodobacter capsulatus* (*dct*PQM) [30] and *Sinorhizobium meliloti* (*mat*PQM) [31], respectively. The results showed that Mae I from *S. pombe* displayed the highest transport efficiency, which has reached to 25.67% (Fig. 3A). Moreover, the amounts of malonate affected transport efficiency of Mae I. As depicted in Additional file 2: Fig. S1, transport efficiency reached the highest value when the concentration of malonate was 60 mM. Thus, this content was used in following studies.

In the malonate metabolism pathway, the second key enzyme is malonyl-CoA synthase, which is responsible for the conversion of malonate to malonyl-CoA. To obtain the appropriate MatB in *S. cerevisiae*, we expressed MatB from three originates through plasmid pRS41H, including *Rp*MatB from *Rhodopseudomonas palustris* [32], *Bj*MatB from *Bradyrhizobium japonicum* [13] and *At*MatB from *Arabidopsis thaliana* [33]. As shown in Fig. 3B, quantitative real-time PCR (qRT-PCR) analysis revealed that mRNA transcription levels of *Bj*MatB and *Rp*MatB genes was highly expressed in these strains over the *At*MatB strain. In vivo enzyme activity assay further determined the superior performance of *Rp*MatB in yeast (Fig. 3C), which was corresponding to our previous results in bacteria [17]. Thus, *Rp*MatB was chosen for subsequent study based on the results of systematically determination at transcription and in vivo enzymatic activity levels.

To produce our targets from malonate, the third stage is the synthesis of acetoacetyl-CoA from malonyl-CoA via two routes. The first pathway is only one step mediated by ACCS, and the second pathway is two steps mediated by MatA and ERG10. To evaluate the performance of these two pathways in production of β-caryophyllene, two engineered strains were constructed by introducing ACCS and MatA two genes into strain YQ-4 via plasmid harboring Mae I or *Rp*MatB encoding genes, respectively, generating engineered strains MBC and MBA. Compared to strain YQ-4, the level of β-caryophyllene in MBC and MBA strains lifted nearly 1- and 0.7-fold (Fig. 3D), respectively. Besides, only overexpressing ACCS or MatA failed to improve the production of β-caryophyllene in the presence of malonate or not. All of these results demonstrated that the additional of malonic acid assimilation pathway can efficiently promote the production of our target.

### Switching carbon metabolic flux for efficient biosynthesis of β-caryophyllene mediated by MAAC pathway

Although episomal plasmids expression can achieve higher protein yield, the strategy requires induction and selection, thus increasing the production cost and raising the stability concern. To avoid these problems, expression cassettes for constitutive and strong expression of Mae I, MatB and ACCS were integrated into sites of the genome to generate strain YQ-7.

**Fig. 4:**
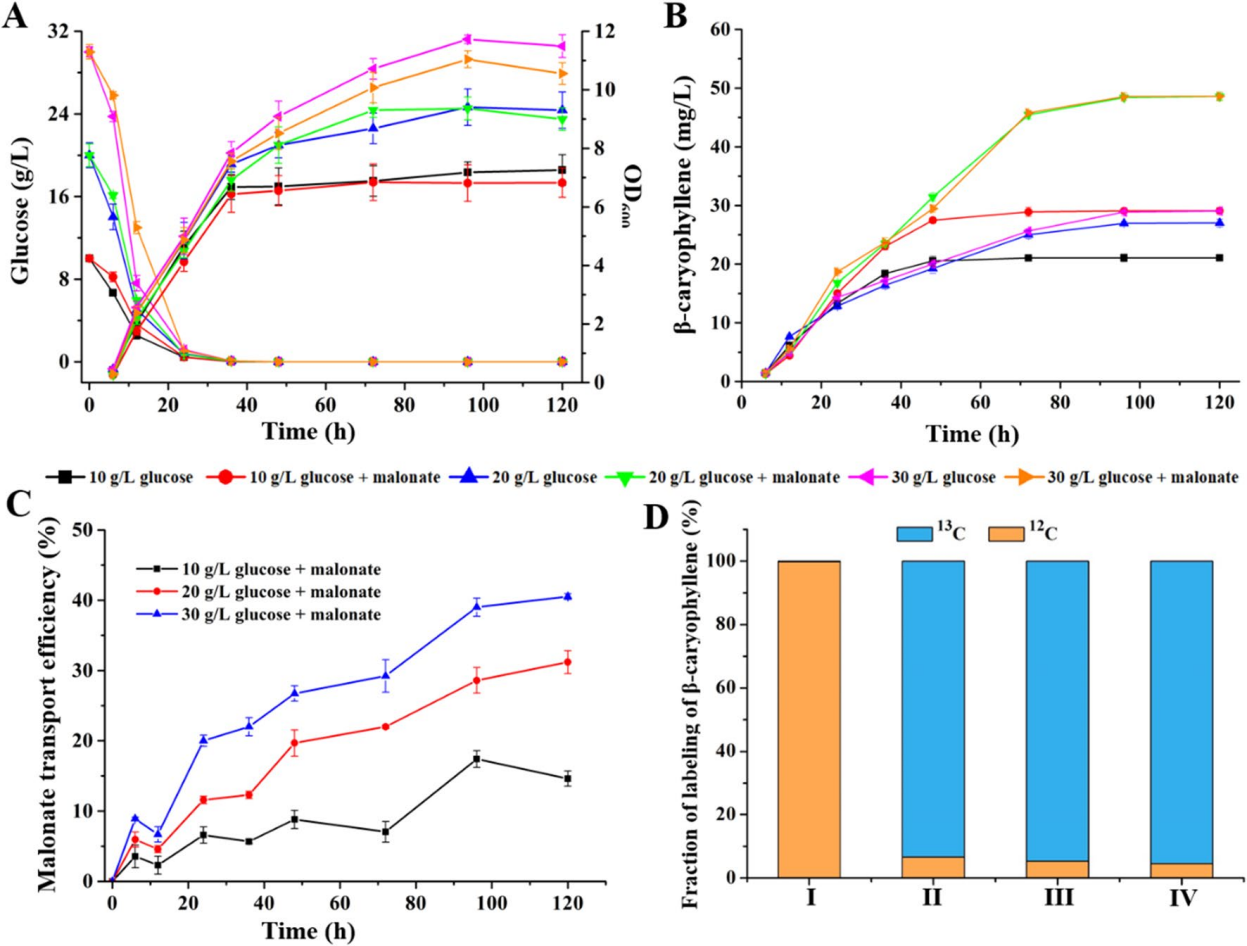
Stable production of β-caryophyllene using engineered yeast YQ-7. **A** Time courses of contents of glucose and cell growth. **B** Time courses of production of β-caryophyllene. **C** Time courses of contents of malonate. **D** Fraction of labeling of β-caryophyllene using different substrates as carbon source. I corresponds to 20 g/L ^12^C-glucose. II corresponds to 10 g/L ^13^C-glucose + 60 mM ^12^C-malonate. III corresponds to 20 g/L ^13^C-glucose + 60 mM ^12^C-malonate. IV corresponds to 30 g/L ^13^C-glucose + 60 mM ^12^C-malonate. Error bars represent the standard deviations of three replicates

To assess the role of MAAC pathway on the production of β-caryophyllene, strain YQ-7 were cultivated in 100-mL shake flask under various amounts of glucose ranging from 10 to 30 g/L with or without a certain content of malonate. As shown in Fig. 4A, C, during the first 12 h, the cells density and the titer were influenced by the addition of malonate. After 12 h’ culture, the titer was gradually improved and reached to 48.56 ± 0.61 mg/L at 120 h when the concentration of glucose was 20 g/L (Fig. 4B), and meanwhile, malonate was also continually transported into cells and transport efficiency attained to about 31.19% at the same time (Fig. 4C). Overall, both the biomass and β-caryophyllene synthesis in YQ-7 were improved with increasing glucose concentration from 10 to 20 g/L. The production capacity improved 67% upon the addition of malonate, whereas only 28% increased level was observed as only feeding glucose to this engineered strain (Fig. 4B). Importantly, relative to only glucose, β-caryophyllene formation was enhanced around 80% when both glucose (20 g/L) and malonate were simultaneously employed (Fig. 4B). When the amounts of glucose up to 30 g/L, this improvement failed to further expand regardless of the component of carbon source (Fig. 4B). Combined with the result shown in Fig. 2, the production of β-caryophyllene increased nearly onefold when using strain YQ-7 as microbial cell factory comparing with YQ-4 strain. This indicated that the metabolic status of yeast cells was altered by recruiting MAAC pathway genes. However, the molecular mechanisms underlying these changes are unknown.

We wished to determine whether improved yields in strain YQ-7 arose via the malonate metabolism pathway. ^13^C‑labeling method was used to quantify the proportion of produced β-caryophyllene from malonate and glucose, respectively. ^13^C-labeled malonate is not commercially available but labeled glucose is. We conducted several carbon labeling experiments providing (i) ^13^C-labeled glucose at different contents with a certain amount of unlabeled malonate; (ii) unlabeled glucose (final concentration of 20 g/L) with a certain amount of unlabeled malonate. After 4 days culture, the ^13^C-labeling patterns of β-caryophyllene were analyzed using GC/MS method. As shown by the labeling pattern observed upon feeding YQ-7 strain with unlabeled glucose and unlabeled malonate, 99.84% of β-caryophyllene unlabeled (Fig. 4D and Additional file 2: Fig. S2). Surprisingly, we observed that the produced β-caryophyllene had ≈94–96% of their carbon labeled, and only ≈4–6% of carbon unlabeled (Fig. 4D and Additional file 2: Fig. S2), suggesting that a small amount of malonate involved in the biosynthesis of our products, and a majority of β-caryophyllene originated from glucose.

In our engineered yeast, acetyl-CoA derived from glucose has three main down-stream pathways, including TCA cycle, fatty acid biosynthesis and β-caryophyllene biosynthesis. Meanwhile, malonate mainly entered into fatty acid biosynthesis route and MVA path for β-caryophyllene biosynthesis in yeast cells. The β-caryophyllene profiles described above clearly indicate that the direct metabolic pathway for β-caryophyllene biosynthesis was glucose-mediated MVA route rather than malonate-mediated MAAC route. Therefore, we hypothesized that the introduction of malonic acid assimilation in *S. cerevisiae* could strengthen the production of β-caryophyllene by reducing the level of carbon flow from acetyl-CoA to malonyl-CoA, resulting in increasing the metabolic flux in MVA route. To test these possibilities and investigate the molecular mechanisms underlying improvement of β-caryophyllene synthesis in yeast, we examined expression of genes involving native metabolisms and engineered pathways under malonate plus glucose vs. only glucose-containing medium in strain YQ-7.

**Fig. 5:**
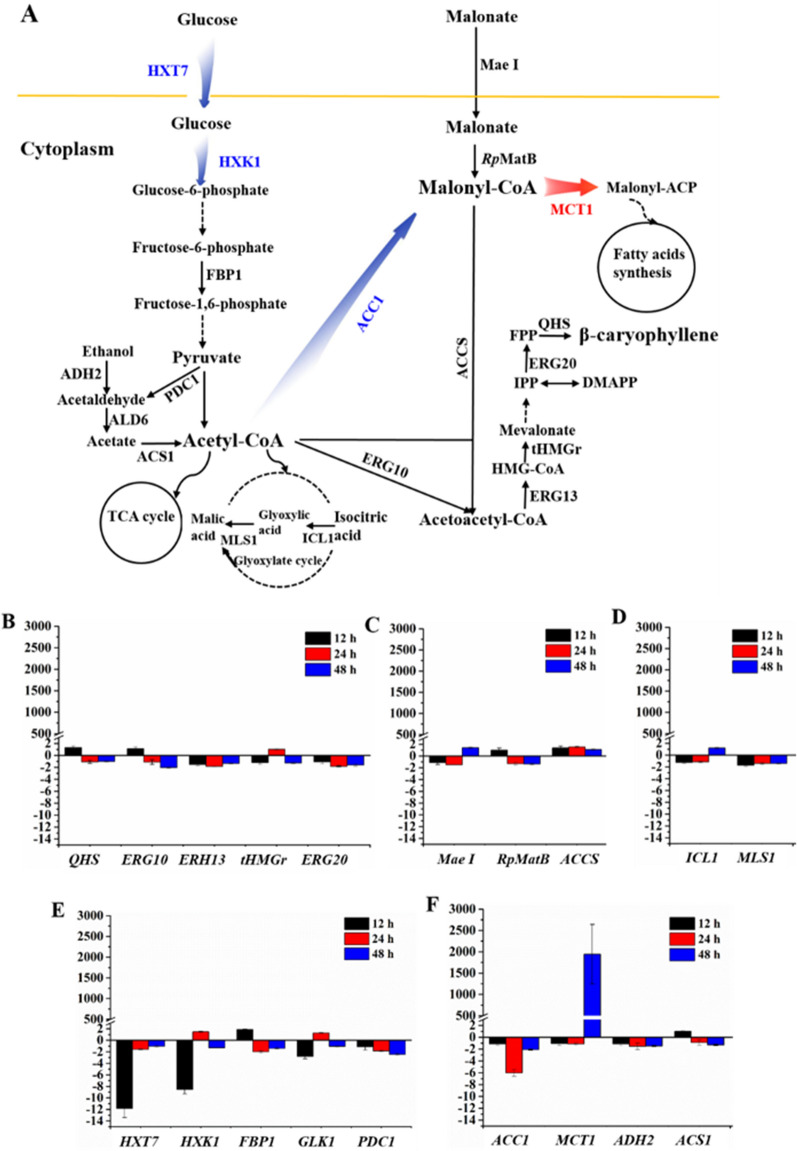
The expression levels of strain YQ-7 feeding on both glucose and malonate were determined relative to control (without malonate) for several genes.** A** The main carbon metabolic network investigated in transcription analysis. Carbon metabolic flux was switched from fatty acid synthesis to β-caryophyllene synthesis mediated by malonic acid assimilation. The mRNA levels of *HXT7*, *HXK1* and *ACC1* genes decreased upon the addition of malonate (blue arrows and blue gene names). The mRNA levels of MCT1 genes boosted considerably after adding malonate (red arrow and red gene name). The profile of other genes expression levels showed no obvious difference with or without malonate (black arrows and black gene names). **B** Genes related to MVA route. **C** Genes related to malonic acid metabolic pathway. **D** Genes related to glyoxylate cycle. **E** Genes related to glucose metabolism pathway. **F** Genes related to fatty acids metabolism pathways and other routes. The error bars correspond to standard deviation (n = 3). ICL1, isocitrate lyase; MLS1, malate synthase; FBP1, fructose-1,6-bisphosphatase; MCT1, malonyl-CoA:acyl-carrier-protein transferase

The results indicated that upon the addition of malonate, there was no obvious change in genes related to native or foreign engineered pathways, including heterogeneous MVA and MAAC routes, TCA cycle, fatty acids degradation process, and even glyoxylate cycle (Fig. 5A, B and C). Prior to the transcription analysis, it was supposed that gene encoding MatB was upregulated in the presence of malonate in recombinant cells. However, the experiment results were inconsistent with our prediction. This gene expression level showed negligible change, indicating that the enzymatic activity of MatB in cells may be sufficient to convert malonate into malonyl-CoA. Strikingly, the addition of malonate to cultures caused obvious decreases in the transcript levels of genes involved in glucose transportation and EMP pathway during the initial 12 h (Fig. 5D). After that, the transcript levels of these genes retained a regular level (Fig. 5D). The addition of malonate also exerted a significant effect on the expression of native ACC1, and up to about sixfold transcriptional down-regulation was observed at 24 h (Fig. 5E), suggesting the carbon flux from acetyl-CoA to malonyl-CoA was sharply reduced in cells. On the contrary, an approximately 1900-fold transcriptional up-regulation of MCT1 was detected at 48 h (Fig. 5E), which is responsible for native biosynthesis of fatty acids. Taken together, all of these results were consistent with our fermentation and ^13^C-labeling experiments results described above, implying the recruitment of malonic acid may efficiently weaken metabolic flux originated from glucose to fatty acids since malonic acid metabolic pathway provided sufficient precursor malonyl-CoA for fatty acids biosynthesis.

### Optimization of fermentation conditions in shake-flask conditions for β-caryophyllene overproduction

**Fig. 6:**
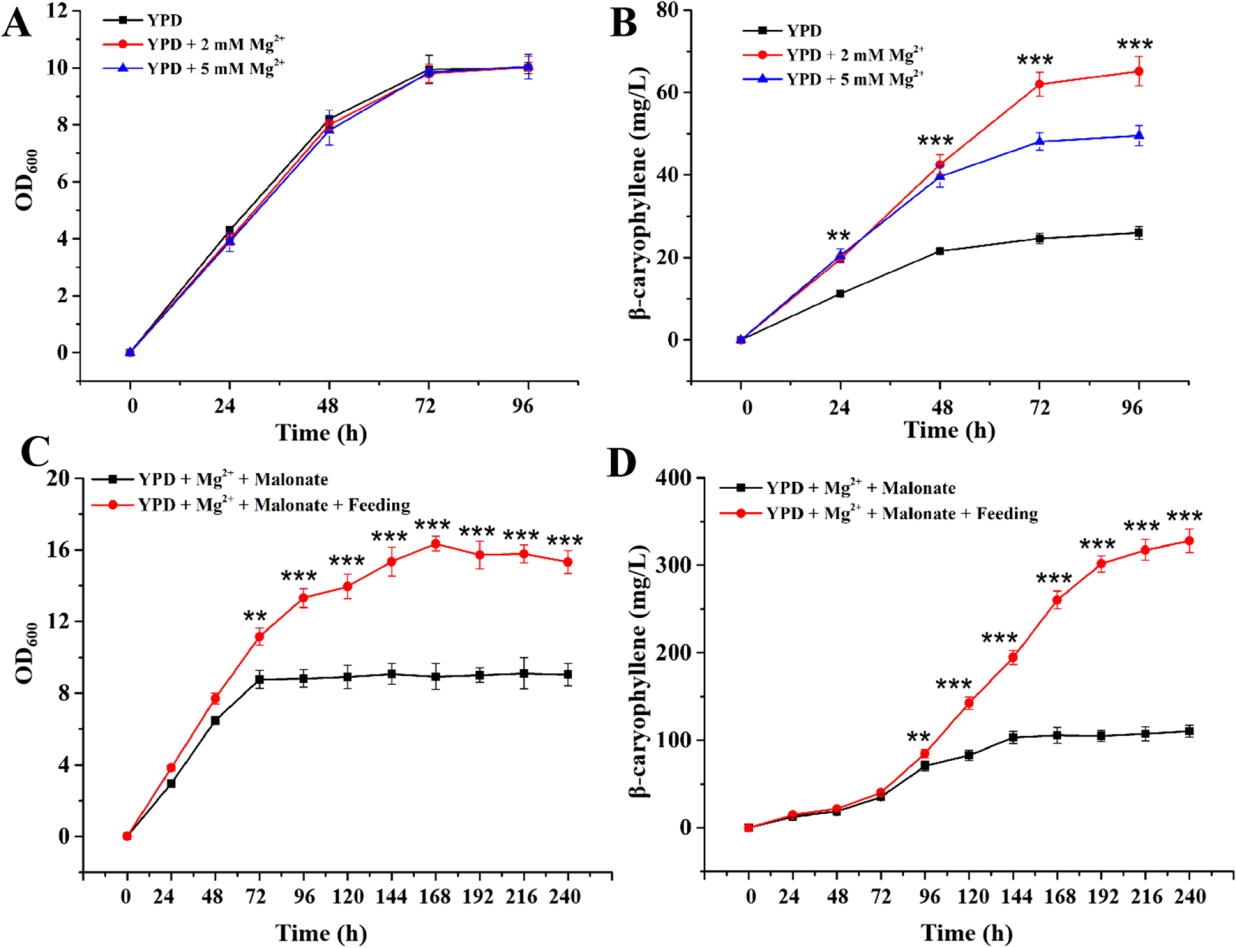
Optimization of fermentation conditions of strain YQ-7. Time courses of cell growth (**A, C)** and β-caryophyllene production (**B, D)** during shake-flask fermentation in culture medium with different components and concentrations were recorded. Error bars indicate the standard deviations of three biological replicates. ** indicates significant pairwise differences between two bars, *p* < 0.01, *** indicates significant pairwise differences between two bars, *p* < 0.001

Here, Mg^2+^ concentrations were optimized to find the most appropriate amount of Mg^2+^ for efficient production of β-caryophyllene. Considering metabolic disturbance induced by malonate, only Mg^2+^ was added to YPD medium for strain YQ-7 growth. As shown in Fig. 6A, the growth curves of strains were similar, indicating that this divalent metal ion showed little influence on cell proliferation; whereas, the addition of Mg^2+^ was found be positively affecting β-caryophyllene production level, and 1.6-fold improved titer was realized when 2 mM of Mg^2+^ was added to YPD medium (Fig. 6B).

To further boost β-caryophyllene production, the shake-flask fermentation process was optimized employing intermittent feeding strategy. Herein, YPD medium containing 2 mM of Mg^2+^ and 60 mM of malonate was used to culture the engineered *S. cerevisiae* YQ-7 in the initial time. When feeding solution containing glucose was fed to the final concentration of 20 g/L at 24, 48, 72, 96, 120 and 144 h during flask cultivation process, the β-caryophyllene producer accumulated up to 328.00 ± 13.44 mg/L, which improved by twofold compared to the control without glucose feeding and 13-fold higher than that of initial titer (25.8 ± 0.7 mg/L) (Fig. 6D). Although cell density increased synchronously, this extent (increased about 70%) was much smaller than that of β-caryophyllene titer (Fig. 6C), suggesting the additional supply of glucose was mainly used for product synthesis instead of cell growth. Besides, the residues of malonate were determined to be 22.95 ± 0.38 mM at 144 h when glucose was fed into cultures, which was 62% of this substrate was consumed during the whole fermentation period; whereas, only ~ 34% of supplied malonate was used by strain YQ-7 without glucose feeding.

To demonstrate the applicability of MAAC metabolic pathway in biosynthesis of other sesquiterpene, the construction of yeast cell factory for β-elemene was conducted. β-Elemene is a volatile sesquiterpenoid natural products and an anti-tumor active ingredient extracted and isolated from traditional Chinese medicine Curcuma [34]. Although biosynthesis of β-elemene in *S. cerevisiae* has been realized in previous studies [35, 36], the application of plasmid expression approach generally has robust and economical concerns. In the present work, to realize stable and controllable production of β-elemene in *S. cerevisiae* CEN.PK2-1D, the QHS encoding gene in strain YQ-7 was replaced by the germacrene A synthase gene GAS by Cas9-based genome editing method to generate strain YQ-8. As shown in Additional file 2: Fig. S3, the target production boosted to 984.36 ± 50.31 mg/L by employing the similar fermentation conditions.

## Discussion

Acetyl-CoA is the basic precursor for sugar and fatty acid metabolisms, and even biosynthesis of isoprenoids. In the previous studies, excessive production of sesquiterpenes could be achieved by increasing the supply of acetyl-CoA [11, 12]. Unfortunately, the titer of sesquiterpenes is still difficult to reach the level of industrial production. In our previous study, we adopted special malonic acid metabolic pathway to achieve the highly efficient supply of malonyl-CoA for producing 3-HP in *E. coli* [17]. Herein, expanding the applications of malonic acid assimilation strategy in biosynthesis of malonyl-CoA derived compounds to other valuable isoprenoids in yeast was attempted. To realize stable and controllable production of β-caryophyllene in *S. cerevisiae* CEN.PK2-1D, genome has been reconstructed by Cas9-based genome editing method. Initially, QHS from *A. annua* was introduced into *S. cerevisiae* CEN.PK2-1D due to its key role in synthesis process of β-caryophyllene. Subsequently, conventional strategy was carried out to improve the production of germacrene A, concentrating upon overexpressing heterogenous or native MVA pathway genes, such as tHMGr, ERG10, ERG20 and ERG13. Consistent with our expectations, the titer was lifted from 7.75 ± 0.01 mg/L to 25.80 ± 0.70 mg/L.

To further strengthen the biosynthesis of isoprenoids in strain YQ-4, malonic acid assimilation pathway was introduced into this engineered *S. cerevisiae*. Malonate transporter and MatB were systematically determined at transcription and in vivo enzymatic activity levels, and Mae I and RpMatB were chosen as the highest efficient enzymes used to produce β-caryophyllene from malonate. In addition to these two steps, the enzymes responsible for conversion of malonyl-CoA to acetoacetyl-CoA were also optimized. The results revealed that ACCS-mediated route was much efficient than MatA-ERG10-mediated way. However, the performance of ACCS for bioproduction of farnesene was unsatisfactory probably due to the insufficiency of malonyl-CoA in cells [25]. Thus, the obvious reinforcement of isoprenoids production in current study was benefited from providing abundant malonyl-CoA for biosynthesis of germacrene A and fatty acids. Shake-flask tests were carried out to determine the effects of the synthetic MAAC pathway in isoprenoids bioproduction. To systematically uncover the underlying molecular mechanism, ^13^C isotopic tracer analysis and quantitative RT-PCR assay were conducted to investigate the role of MAAC pathway in biosynthesis of germacrene A and fatty acids. Taken together, we speculated a tuned mechanism by with malonic acid induced the enhanced biosynthesis of β-caryophyllene. Initially, intracellular malonic acid transported by the action of Mae I may have inhibition influences on glucose transport and metabolism due to its toxicity to cell [28], resulting in limited β-caryophyllene synthesis. Then, with the conversion of malonic acid to malonyl-CoA catalyzed by MatB in cytoplasm, the inhibition was relieved and thus the glucose transportation and metabolism returned to normal level, leading to highly efficient production of β-caryophyllene. During which, the great majority of malonyl-CoA derived from malonate was recruited to produce fatty acids, and this precursor was sufficient for fatty acids synthesis process. Thus, more acetyl-CoA generated from glucose entered into MVA pathway to synthesize the product rather than fatty acids synthesis route. Although malonic acid failed to directly contribute the synthesis of the target, its assimilation in yeast efficiently rewrote the central carbon metabolism network, which was benefit to switch carbon flux towards β-caryophyllene from other metabolites.

In addition to the pathway modification, fermentation condition was another vital point for high-level production of sesquiterpenes in this engineered yeast. It was reported that the isoform of QHS (*AaCPS1*) contains highly conserved DDxxD and RxR motifs, responsible for Mg^2+^ ion-substrate binding [24]. So, Mg^2+^ was probably essential to catalytic activity of QHS due to 99% identity between *AaCPS1* and QHS [24]. Results showed that the addition of Mg^2+^ could strengthen the production of β-caryophyllene by improving the catalytic activity of QHS, which was consistent with previous efforts for producing other sesquiterpenes, such as patchoulol [37]. As well known, feeding modes is a very important factor for high-level of production. We found that the higher initial sugar supply only led to increase in biomass rather than production capability as described above. Thus, intermittent feeding strategy was attempted. After 144 h with 6 times supplement of glucose, the highest β-caryophyllene titer of 328.00 ± 13.44 mg/L was obtained, which was 13-fold higher than that of initial titer (25.8 ± 0.7 mg/L). The yield of YQ-7 from glucose to β-caryophyllene was 2.2 mg/g. The present titer of β-caryophyllene in engineered *S. cerevisiae* YQ-7 was 2- and 26-fold higher than the titers through adaptive evolution and engineering β-alanine metabolism in other *S. cerevisiae*, respectively [21, 22]. As another sesquiterpenes, the biosynthesis of β-elemene has attracted great attentions [35, 36, 38]. Similarly, the improvement of the production of β-elemene by introducing MAAC metabolic pathway in yeast was also appropriate. The titer of 984.36 ± 50.31 mg/L in YQ-8 was 2- to 4-fold higher than the titers in *S. cerevisiae* in previous studies [35, 36]. Moreover, the difference of consumed malonate amounts for two fermentation conditions implying the persistent effects mediated by MAAC pathway for production of our target. Therefore, these results confirmed our proposed mechanism described above. MAAC pathway can continually provide sufficient malonyl-CoA for native metabolism. So provided glucose concentrated on the biosynthesis of sesquiterpenes.

## Conclusions

In conclusion, we have demonstrated the potential of a synthetic MAAC pathway to synthesize sesquiterpene-based high-density biofuel precursor in *S. cerevisiae* in this study. The mechanism by with malonic acid induced the promotive biosynthesis of β-caryophyllene has been studied in both transcriptional and metabolic levels, which is strains with rewired central metabolism can devote more sugars to target production. Finally, the yeast strain accumulated β-caryophyllene at the highest levels in a flask fermentation under optimum conditions. To the authors’ best knowledge, this is the first report on the stable and cost-efficient bioproduction of β-caryophyllene via MAAC pathway with the highest titers obtained by yeast cell factories. This study broadens the application of MAAC pathway in the biosynthesis of high-value natural products, especially sesquiterpene-based high-density biofuel precursor β-caryophyllene.

## Materials and methods

### Plasmid and strain construction

Plasmids and strains are listed in Additional file 1: Table S1. The protein sequences of *QHS*, tHMGr, IDI, ERG20, ERG10, ERG13, *Rl*MatC, Mae I, MdcF, MadLM, *dct*PQM, *mat*PQM, *Rp*MatB, *Bj*MatB, *At*MatB, MatA, ACCS and *Ls*GAS were obtained from the NCBI database (accession numbers: *QHS*, AAL79181.1; tHMGr, YP_164994; IDI, QHB12144; ERG20, QHB09467; ERG10, QHB12229; ERG13, QHB10613; *Rl*MatC, ACI538721; Mae I, AAC49149; MdcF, AAC45458; MadLM, ENV54285 and ENV54286; *dct*PQM,CAA45387, CAA45386 and CAA45385; *mat*PQM, ABR63913, AAK64740 and ABR63911; *Rp*MatB, CAE25665; *Bj*MatB, AAF28840; *At*MatB, OAP02278; MatA and ACCS, BAJ10048; *Ls*GAS, AAM11627). Genes encoding these proteins were codon-optimized for *S. cerevisiae* and synthesized by Beijing Liuhe Huada Gene Corporation (Beijing, China). *Escherichia coli* DH5α was the host strain used for the construction of vector. The strain was cultured in Luria–Bertani (LB) medium (10 g/L peptone, 5 g/L yeast extract and 10 g/L sodium chloride) at 200 rpm and 37 °C. Gene insertions and deletions on the chromosome of *S. cerevisiae* CEN.PK2-1D (*MatALPHA; ura3-52, trp1-289, leu2-3,112, his3∆1; MAL2-3c; SUC2*) were conducted using CRISPR/Cas9 based on homologous recombination [39], and transformation of yeast cells was carried out using a lithium acetate method. In detail, the CRISPR–Cas9 system with double-plasmid system derived from the *Streptococcus pyogenes* was adopted, one plasmid for the expression of Cas9 endonuclease and the other for the expression of guide-RNA with 20-bp targeting sequence. When deleting open reading frames, we used 70 bp immediately upstream of the start codon and 70 bp immediately downstream of the stop codon as regions of homology for construct integration, unless otherwise specified. For all parts native to *S. cerevisiae*, we used genomic DNA from CEN.PK2-1D as a template for PCR amplification. Primers used in polymerase chain reaction (PCR) in this work are listed in Additional file 1: Table S2. The enzymes and kits used for molecular cloning were obtained from Vazyme Biotech Co., Ltd, China. Yeast strains were grown in Yeast Extract Peptone Dextrose (YPD) medium (10 g/L yeast, 20 g/L peptone and 20 g/L glucose) at 30 °C with shaking at 200 rpm. Whenever necessary, antibiotics were added as follows: ampicillin (100 μg/mL), Hygromycin B (100 μg/mL) and Geneticin (100 μg/mL).

### Shake-flask experiments

All of engineered strains were grown in 5 mL of YPD medium at 30 °C and 200 rpm for 16 h. Then cells were diluted 100-fold in 100 mL fresh YPD medium and regrown with shaking 30 °C. For strains harboring malonate metabolic genes, malonate was added into medium at the same time. For β-caryophyllene and β-elemene analysis, 10% (*v*/*v*) dodecane was also added into the culture to capture the products in the organic layer, respectively.

### Analytical methods

The determination of malonic acid and malonyl-CoA was performed according to our previous work [17]. The content of glucose was determined by 3,5-dinitrosalicylic acid colorimetry (DNS method). The production of β-caryophyllene and β-elemene was quantified using an Agilent Technologies 7890B Gas Chromatograph equipped with a flame ionization detector (FID), respectively. β-Caryophyllene (Sigma-Aldrich, US) was used as the standard compound to construct the standard curve for the production of β-caryophyllene, and the standard curve for β-elemene was prepared using the β-elemene of Sigma-Aldrich(US) as the standard substance. Each analyte from the 1 μL samples was separated on a 19091 J HP-5 column (length, 30 m; internal diameter, 0.32 mm; film thickness, 250 mm). The column temperature profile of β-caryophyllene was: rate 1 °C/min from 54 °C to a final temperature of 65 °C, then rate 10 °C/min to a final temperature of 115 °C, rate 1 °C/min to a final temperature of 120 °C, rate 0.5 °C/min to a final temperature of 125 °C, rate 80 °C/min to a final temperature of 280 °C, held for 7 min. The GC oven temperature program of β-elemene was: 80 °C for 1 min, increasing the temperature with a heating rate of 25 °C/min up to 180 °C, held for 5 min, and then increasing the temperature with a heating rate of 15 °C/min up to 280 °C, held for 3 min. Nitrogen was used as the carrier gas at a flow rate of 1.0 mL/min. The detector temperature was maintained at 300 ℃ for the determination of β-caryophyllene and 260 ℃ for the determination of β-elemene, respectively.

### ^13^C isotopic tracer analysis

The recombinant yeast strains were grown on YPD agar medium containing fully ^13^C-labeled glucose (purchased from Sigma Aldrich, USA). Single clone was grown in 5 mL of YPD medium containing fully ^13^C-labeled glucose at 30 °C and 200 rpm for 16 h. Then cells were diluted 100-fold in 100 mL fresh YPD medium containing fully ^13^C-labeled glucose and ^12^C-labeled malonate and regrown with shaking 30 °C for 72 h. For β-caryophyllene analysis, 10% (*v*/*v*) dodecane was also added into the culture to capture the products. The samples from the organic layer were taken to detect the generated β-caryophyllene by GC/MS approach. The analysis was performed with a SHIMADZU GCMS-QP2020 system in scan mode. The SH-I-5Sil MS chromatography column was used. The GC oven temperature program was: 100 °C for 1 min, rate 10 °C/min to a final temperature of 220 °C, held for 2 min. Helium was used as a carrier gas at a flow rate of 1.2 mL/min. The injector was operated in splitless mode. The MSD parameters were set as EI at 70 eV, mass range of 50–500 Da, and the scan speed at 2 scans/s.

### Quantitative RT-PCR assay

To conduct transcription analysis of malonyl-CoA synthase from different sources, strains harboring genes were grown in YPD medium for 12 h at 30 °C and 200 rpm. Cells were centrifuged at 4000 × g and 4 °C for 10 min followed by quick-frozen in liquid nitrogen. RNA was extracted by HiPure Yeast RNA kit according to the manufacturer’s instructions (Magen, China). The cDNA synthesis kit was used for reverse transcription (Takara Company, Japan). The ChamQ Universal SYBR qPCR Master Mix (Vazyme Biotech Co., Ltd, China) and the qTOWER^3^ Real-Time PCR Thermal Cycler (Jena, Germany) were used for all quantitative assays. Constitutively transcribed *Alg9* was used as a reference control to normalize the total RNA quantity of different samples. The primers used for the assays are listed in Additional file 1: Table S3. The comparative *C*_*T*_ method was used for relative quantification of gene expression. The relative quantification for each of mRNA levels was calculated using the Δ*C*_*T*_ method, which is the difference between the *C*_*T*_ of the gene of interest and that of the reference gene.

To investigate perturbation of gene expression induced by the addition of malonate to yeast culture, YQ-7 strain was grown in YPD medium with or without malonate at 30 °C and 200 rpm. The engineered strains were sampled after 12 h, 24 h and 48 h cultivation, respectively. Then, the samples were centrifuged at 4000 × g and 4 °C for 10 min followed by quick-frozen in liquid nitrogen. qRT-PCR analysis of interest genes was carried out as described above. The primers used for the assays are listed in Additional file 1: Table S3. The relative difference of mRNA level was calculated using the ΔΔ*C*_*T*_ method, which is the difference Δ*C*_*T*_ of the gene of interest in the presence of malonate and that of gene in the absence of malonate. Three independent biological samples with three technical repeats for each sample were performed for qRT-PCR analysis.

### Optimization of flask fermentation process

For flask fermentation experiments, seed cultures were prepared by culturing in 5 mL of YPD medium containing 20 g/L glucose overnight. Yeast cells were then diluted 100-fold and inoculated into 100 mL YPD medium with 20% glucose in 250 mL flask at 30 °C and 200 rpm. Dodecane (10 vol %) was added in initial time for extracting products. To explore the effects of divalent metal ion on β-caryophyllene production, Mg^2+^ was added into YPD medium at two concentrations, including 2 mM and 5 mM, respectively. For achieve high-level production of β-caryophyllene, intermittent feeding strategy was attempted. A single clone picked from the YPD plate was grown in 5 mL YPD medium for 12 h, and the culture was diluted 100-fold into a 250-mL shake-flask containing 100 mL of YPD with 20 g/L glucose, 2 mM Mg^2+^ as well as 10% dodecane (*v*/*v*) at 30 °C and 200 rpm. A concentrated solution containing 60% glucose (*v*/*v*) was fed into cultures in an intermittent way. Biomass, extracellular β-caryophyllene titer and residual malonate were monitored during the whole process.

### Supplementary Information

**Additional file 1: Table S1.** Strains and plasmids used in this study. **Table S2.** All primers for gene amplification used in this study. **Table S3.** All primers used in transcription analysis of genes

**Additional file 2: Figure S1.** Malonate transport efficiency of strain Mae I with different concentrations of malonate. **Figure S2.** Detection of β-caryophyllene by GC–MS in ^13^C isotopic tracer assay. (A) The total ion chromatogram of β-caryophyllene. (B) The mass spectrum of the β-caryophyllene peak in (A). **Figure S3.** Time courses of cell growth (A) and β-elemene production (B) during shake-flask fermentation in culture medium under different fermentation conditions. Error bars indicate the standard deviations of three biological replicates.

## Data Availability

All data generated or analyzed during this study are included in this published article (and its Additional files).

## References

[1] Newman J, Bonino CA, Trainham JA (2018). The energy future. Annu Rev Chem Biomol Eng.

[2] Athanasakoglou A, Kampranis SC (2019). Diatom isoprenoids: advances and biotechnological potential. Biotechnol Adv.

[3] Beller HR, Lee TS, Katz L (2015). Natural products as biofuels and bio-based chemicals: fatty acids and isoprenoids. Nat Prod Rep.

[4] Harvey BG, Meylemans HA, Gough RV, Quintana RL, Garrison MD, Bruno TJ (2014). High-density biosynthetic fuels: the intersection of heterogeneous catalysis and metabolic engineering. Phys Chem Chem Phys.

[5] Dahham SS, Tabana YM, Iqbal MA, Ahamed MB, Ezzat MO, Majid AS, Majid AM (2015). The anticancer, antioxidant and antimicrobial properties of the sesquiterpene β-Caryophyllene from the essential oil of *Aquilaria crassna*. Molecules.

[6] Quispe-Condori S, Foglio MA, Rosa PTV, Angela M, Meireles A (2008). Obtaining β-caryophyllene from *cordia verbenacea de candolle* by supercritical fluid extraction. J Supercrit Fluid.

[7] Topal U, Sasaki M, Goto M, Otles S (2008). Chemical compositions and antioxidant properties of essential oils from nine species of Turkish plants obtained by supercritical carbon dioxide extraction and steam distillation. Int J Food Sci Nutr.

[8] Yang JM, Li ZF, Guo LZ, Du J, Bae HJ (2016). Biosynthesis of beta-caryophyllene, a novel terpene-based high-density biofuel precursor, using engineered *Escherichia coli*. Renew Energy.

[9] Navale GR, Dharne MS, Shinde SS (2021). Metabolic engineering and synthetic biology for isoprenoid production in *Escherichia coli* and *Saccharomyces cerevisiae*. Appl Microbiol Biot.

[10] Zhang CB, Liu JJ, Zhao FL, Lu CZ, Zhao GR, Lu WY (2018). Production of sesquiterpenoid zerumbone from metabolic engineered *Saccharomyces cerevisiae*. Metab Eng.

[11] Meadows AL, Hawkins KM, Tsegaye Y, Antipov E, Kim Y, Raetz L, Dahl RH, Tai A, Mahatdejkul-Meadows T, Xu L (2016). Rewriting yeast central carbon metabolism for industrial isoprenoid production. Nature.

[12] Daletos G, Katsimpouras C, Stephanopoulos G (2020). Novel strategies and platforms for industrial isoprenoid engineering. Trends Biotechnol.

[13] Kim YS (2002). Malonate metabolism: biochemistry, molecular biology, physiology, and industrial application. J Biochem Mol Biol.

[14] Chae TU, Ahn JH, Ko YS, Kim JW, Lee JA, Lee EH, Lee SY (2020). Metabolic engineering for the production of dicarboxylic acids and diamines. Metab Eng.

[15] Wang YC, Chen H, Yu O (2014). A plant malonyl-CoA synthetase enhances lipid content and polyketide yield in yeast cells. Appl Microbiol Biot.

[16] Park SR, Ahn MS, Han AR, Park JW, Yoon YJ (2011). Enhanced flavonoid production in *Streptomyces venezuelae* via metabolic engineering. J Microbiol Biotechnol.

[17] Liang B, Sun GN, Wang ZB, Xiao J, Yang JM (2019). Production of 3-hydroxypropionate using a novel malonyl-CoA-mediated biosynthetic pathway in genetically engineered E. coli strain. Green Chem.

[18] Sui X, Zhao M, Liu Y, Wang J, Li G, Zhang X, Deng Y (2020). Enhancing glutaric acid production in *Escherichia coli* by uptake of malonic acid. J Ind Microbiol Biotechnol.

[19] Deng XM, Shi B, Ye ZL, Huang M, Chen R, Cai YS, Kuang ZL, Sun X, Bian GK, Deng ZX, Liu TG (2022). Systematic identification of ocimum sanctum sesquiterpenoid synthases and (-)-eremophilene overproduction in engineered yeast. Metab Eng.

[20] Li MJ, Hou FF, Wu T, Jiang XL, Li FL, Liu HB, Xian M, Zhang HB (2020). Recent advances of metabolic engineering strategies in natural isoprenoid production using cell factories. Nat Prod Rep.

[21] Godara A, Kao KC (2021). Adaptive laboratory evolution of beta-caryophyllene producing *Saccharomyces cerevisiae*. Microb Cell Fact.

[22] Lu S, Zhou C, Guo X, Du Z, Cheng Y, Wang Z, He X (2022). Enhancing fluxes through the mevalonate pathway in *Saccharomyces cerevisiae* by engineering the hmgr and β-alanine metabolism. Microb Biotechnol.

[23] Cheng T, Zhang K, Guo J, Yang Q, Li YT, Xian M, Zhang RB (2022). Highly efficient biosynthesis of beta-caryophyllene with a new sesquiterpene synthase from tobacco. Biotechnol Biofuels Bioproducts.

[24] Muthusamy S, Vetukuri RR, Lundgren A, Ganji S, Zhu LH, Brodelius PE, Kanagarajan S (2020). Transient expression and purification of beta-caryophyllene synthase in nicotiana benthamiana to produce beta-caryophyllene in vitro. PeerJ.

[25] Tippmann S, Ferreira R, Siewers V, Nielsen J, Chen Y (2017). Effects of acetoacetyl-CoA synthase expression on production of farnesene in *Saccharomyces cerevisiae*. J Ind Microbiol Biotechnol.

[26] Sumegi B, Sherry AD, Malloy CR (1990). Channeling of TCA cycle intermediates in cultured *Saccharomyces cerevisiae*. Biochemistry.

[27] Chen WN, Tan KY (2013). Malonate uptake and metabolism in *Saccharomyces cerevisiae*. Appl Biochem Biotechnol.

[28] Hoenke S, Schmid M, Dimroth P (2000). Identification of the active site of phosphoribosyl-dephospho-coenzyme A transferase and relationship of the enzyme to an ancient class of nucleotidyltransferases. Biochemistry.

[29] Stoudenmire JL, Schmidt AL, Tumen-Velasquez MP, Elliott KT, Laniohan NS, Walker Whitley S, Galloway NR, Nune M, West M, Momany C (2017). Malonate degradation in *Acinetobacter baylyi ADP1*: operon organization and regulation by MdcR. Microbiology.

[30] Kelly DJ, Thomas GH (2001). The tripartite ATP-independent periplasmic (TRAP) transporters of bacteria and archaea. FEMS Microbiol Rev.

[31] Chen AM, Wang YB, Jie S, Yu AY, Luo L, Yu GQ, Zhu JB, Wang YZ (2010). Identification of a TRAP transporter for malonate transport and its expression regulated by GtrA from *Sinorhizobium meliloti*. Res Microbiol.

[32] Crosby HA, Rank KC, Rayment I, Escalante-Semerena JC (2012). Structure-guided expansion of the substrate range of methylmalonyl coenzyme A synthetase (MatB) of *Rhodopseudomonas palustris*. Appl Environ Microbiol.

[33] Chen H, Kim HU, Weng H, Browse J (2011). Malonyl-CoA synthetase, encoded by acyl activating enzyme13, is essential for growth and development of *Arabidopsis*. Plant Cell.

[34] Zhai BT, Zhang NN, Han XM, Li QJ, Zhang MM, Chen XY, Li GH, Zhang RN, Chen P, Wang WG (2019). Molecular targets of beta-elemene, a herbal extract used in traditional Chinese medicine, and its potential role in cancer therapy: a review. Biomed Pharmacother.

[35] Zhang W, Guo J, Wang Z, Li Y, Meng X, Shen Y, Liu W (2021). Improved production of germacrene A, a direct precursor of β-elemene, in engineered *Saccharomyces cerevisiae* by expressing a cyanobacterial germacrene A synthase. Microb Cell Fact.

[36] Hu Y, Zhou YJ, Bao J, Huang L, Nielsen J, Krivoruchko A (2017). Metabolic engineering of *Saccharomyces cerevisiae* for production of germacrene A, a precursor of beta-elemene. J Ind Microbiol Biotechnol.

[37] Liu M, Lin YC, Guo JJ, Du MM, Tao XY, Gao B, Zhao M, Ma YS, Wang FQ, Wei DZ (2021). High-level production of sesquiterpene patchoulol in *Saccharomyces cerevisiae*. ACS Synth Biol.

[38] Ye M, Gao J, Zhou YJ (2023). Global metabolic rewiring of the nonconventional yeast *Ogataea polymorpha* for biosynthesis of the sesquiterpenoid beta-elemene. Metab Eng.

[39] Zhang GC, Kong II, Kim H, Liu JJ, Cate JHD, Jin YS (2014). Construction of a quadruple auxotrophic mutant of an industrial polyploid *Saccharomyces cerevisiae* strain by using RNA-guided cas9 nuclease. Appl Environ Microb.

