# Supplementary material for: Switching carbon metabolic flux for enhancing the production of sesquiterpene-based high-density biofuel precursor in Saccharomyces cerevisiae
Source: Biotechnol Biofuels Bioprod. 2023 Aug 4;16:124. doi: 10.1186/s13068-023-02370-8 (PMC10403917; doi:10.1186/s13068-023-02370-8)
Supplement: Supplementary file 1 — Additional file 1: Table S1. Strains and plasmids used in this study. Table S2. All primers for gene amplification used in this study. Table S3. All primers used in transcription analysis of genes [file 13068_2023_2370_MOESM1_ESM.docx]

**Switching carbon metabolic flux for enhanced production of sesquiterpene-based high-density biofuel precursor in engineered yeast**

Bo Liang^a,b^, Qun Yang^a,b^, Xinping Zhang^a,b^, Yukun Zhao^c^, Yunhui Liu^a,b^, Jianming Yang^a,b^*, Zhaobao Wang^a,b^*

^a^Energy-rich Compounds Production by Photosynthetic Carbon Fixation Research Center, Qingdao Agricultural University, Qingdao, China.

^b^Shandong Key Lab of Applied Mycology, College of Life Sciences, Qingdao Agricultural University, Qingdao, China

^c^Pony Testing International Group, Qingdao, China

^*^Correspondence: [yjming888@126.com](mailto:yjming888@126.com), [wangzhaobao123@126.com](mailto:wangzhaobao123@126.com)

Bo Liang, Qun Yang and Xinping Zhang contributed equally to this work

**Table S1** Strains and plasmids used in this study.

| Name | Description | Reference |
| --- | --- | --- |
| **Strains** |  |  |
| *S. cerevisiae* | CEN.PK2-1D (*MatALPHA; ura3-52, trp1-289, leu2-3,112, his3∆1; MAL2-3c; SUC2*) | Invitrogen |
| YQ-1 | CEN.PK2-1D *ho∆::P**_PGK1_-QHS-T_CYC1_* | This study |
| YQ-2 | CEN.PK2-1D *ho∆::P_PGK1_-QHS-T_CYC1_, ygr250c∆::P_TDH3_-tHMGr-T_HMGR_-T_CYC1_-IDI1-P_CCW12_* | This study |
| YQ-3 | CEN.PK2-1D *ho∆::P_PGK1_-QHS-T_CYC1_, ygr250c∆::P_TDH3_-tHMGr-T_HMGR_-T_CYC1_-IDI1-P_CCW12_, gal80∆::P_CCW12_-ERG20-T_CYC1_-T_TDH3_-ERG10-P_TDH3_* | This study |
| YQ-4 | CEN.PK2-1D *ho∆::P_PGK1_-QHS-T_CYC1_, ygr250c∆::P_TDH3_-tHMGr-T_HMGR_-T_CYC1_-IDI1-P_CCW12_, gal80∆::P_CCW12_-ERG20-P_CYC1_-T_TDH3_-ERG10-P_TDH3_, ndt80∆::P_TDH3_-ERG13-P_TDH3_-T_B_-ERG20-P_PGK1_* | This study |
| YQ-5 | CEN.PK2-1D *ho∆::P_PGK1_-QHS-T_CYC1_, ygr250c∆::P_TDH3_-tHMGr-T_HMGR_-T_CYC1_-IDI1-P_CCW12_, gal80∆::P_CCW12_-ERG20-P_CYC1_-T_TDH3_-ERG10-P_TDH3_, ndt80∆::P_TDH3_-ERG13-T_TDH3_-T_B_-ERG20-P_PGK1_, hxt3∆::P_TDH3_-Mae I-T_TDH3_* | This study |
| YQ-6 | CEN.PK2-1D *ho∆::P_PGK1_-QHS-T_CYC1_, ygr250c∆::P_TDH3_-tHMGr-T_HMGR_-T_CYC1_-IDI1-P_CCW12_, gal80∆::P_CCW12_-ERG20-P_CYC1_-T_TDH3_-ERG10-P_TDH3_, ndt80∆::P_TDH3_-ERG13-P_TDH3_-T_B_-ERG20-P_PGK1_, hxt3∆::P_TDH3_-Mae I-T_TDH3_, gpp1∆::P_TEF1_-ACCS-T_B_* | This study |
| YQ-7 | CEN.PK2-1D *ho∆::P_PGK1_-QHS-T_CYC1_, ygr250c∆::P_TDH3_-tHMGr-T_HMGR_-T_CYC1_-IDI1-P_CCW12_, gal80∆::P_CCW12_-ERG20-P_CYC1_-T_TDH3_-ERG10-P_TDH3_, ndt80∆::P_TDH3_-ERG13-P_TDH3_-T_B_-ERG20-P_PGK1_, hxt3∆::P_TDH3_-Mae I-T_TDH3_, gpp1∆::P_TEF1_-ACCS-T_B_, dit1∆::P_TEF1_-RpMat B-T_CYC1_* | This study |
| YQ-8 | CEN.PK2-1D *ho∆::P_TDH3_-Ls GAS-T, ygr250c∆::P_TDH3_-tHMGr-T_HMGR_-T_CYC1_-IDI1-P_CCW12_, gal80∆::P_CCW12_-ERG20-P_CYC1_-T_TDH3_-ERG10-P_TDH3_, ndt80∆::P_TDH3_-ERG13-P_TDH3_-T_B_-ERG20-P_PGK1_, hxt3∆::P_TDH3_-Mae I-T_TDH3_, gpp1∆::P_TEF1_-ACCS-T_B_, dit1∆::P_TEF1_-RpMat B-T_CYC1_* | This study |
| RlMatC | CEN.PK2-1D/pYQ01 | This study |
| Mae I | CEN.PK2-1D/pYQ02 | This study |
| MdcF | CEN.PK2-1D/pYQ04 | This study |
| MadLM | CEN.PK2-1D/pYQ05 | This study |
| DctPQM | CEN.PK2-1D/pYQ06 | This study |
| MatPQM | CEN.PK2-1D/pYQ07 | This study |
| AtMat B | CEN.PK2-1D/pYQ08 | This study |
| BjMat B | CEN.PK2-1D/pYQ09 | This study |
| RpMat B | CEN.PK2-1D/pYQ10 | This study |
| Mat A | CEN.PK2-1D/pYQ11 | This study |
| ACCS | CEN.PK2-1D/pYQ12 |  |
| MBA | CEN.PK2-1D/pYQ13 | This study |
| MBC | CEN.PK2-1D/pYQ14 | This study |
| **plasmids** |  |  |
| pYQ01 | pRS41H-*P_TDH3_-RlMatC-T_ADH1_* | This study |
| pYQ02 | pRS41H-*P_TDH3_-Mae I-T_ADH1_* | This study |
| pYQ04 | pRS41H-*P_TDH3_-MdcF-T_ADH1_* | This study |
| pYQ05 | pRS41H-*P_TDH3_-MadLM-T_ADH1_* | This study |
| pYQ06 | pRS41H-*P_TDH3_-dctPQM-T_ADH1_* | This study |
| pYQ07 | pRS41H-*P_TDH3_-matPQM-T_ADH1_* | This study |
| pYQ08 | pRS41H-*P_TDH3_-Mae I-T_TDH3_-P_TEF1_-AtMat B-T_ADH1_* | This study |
| pYQ09 | pRS41H-*P_TDH3_-Mae I-T_TDH3_-P_TEF1_-BjMat B-T_ADH1_* | This study |
| pYQ10 | pRS41H-*P_TDH3_-Mae I-T_TDH3_-P_TEF1_-RpMat B-T_ADH1_* | This study |
| pYQ11 | pRS41H-*P_PGK1_-Mat A-T_ADH1_* | This study |
| pYQ12 | pRS41H-*P_PPGK1_-ACCS-T_ADH1_* | This study |
| pYQ13 | pRS41H-*P_TDH3_-Mae 1-T_TDH3_-P_TEF1_-RpMat B-T_ADH1_-T_B_-Mat A-P_PGK1_* | This study |
| pYQ14 | pRS41H-*P_TDH3_-Mae 1-T_TDH3_-P_TEF1_-RpMat B-T_ADH1_-T_B_-ACCS-P_PGK1_* | This study |
| pYQ15 | pRS42H-ho-gRNA | This study |
| pYQ16 | pRS42H-ygr250c-gRNA | This study |
| pYQ17 | pRS42H-gal80-gRNA | This study |
| pYQ18 | pRS42H-ndt80-gRNA | This study |
| pYQ19 | pRS42H-QHS-gRNA | This study |

**Table S2** All primers for gene amplification used in this study.

| **Primer** | **Sequence(5’→3’)** | **Genes** |
| --- | --- | --- |
| 1-PGK1-F | TCATAAGCAGCAATCAATTCTATCTATACTTTAAAGTTTGCAAAAAGAACAAAACTGAA | 1-P_PGK1_ |
| 1-PGK1-R | CTTCTTTAACAGACATTGTTTTATATTTGTTGTAAAAAG |  |
| 1-QHS-F | CAAATATAAAACAATGTCTGTTAAAGAAGAAAAAGTT | 1-QHS |
| 1-QHS-R | GAAAAGGGGCCTGTTTAGATCGGGATCGGGTGAACCAGC |  |
| 1-CYC1-F | CGATCCCGATCTAAACAGGCCCCTTTTCCTTTGTCGAT | 1-T_CYC1_ |
| 1-CYC1-R | TATTACATACAACTTTTTAAACTAATATACACATTAAAAAAAAGAAAAATTTGAAATAT |  |
| 2-PTDH3-F | AATAAACCATAAGTTTTATTTTACTAAAAACATTATACGTGAAAGACAAACCGCATCAGAAGTTTCGAGGAAGACCCGTTGAAAAGAACTTAC | 2-P_TDH3_ |
| 2-PTDH3-R | GGTGGTGGTGCATTTTGTTTGTTTATGTGTGTTTATTCG |  |
| 2-tHMGr-T-F | CATAAACAAACAAAATGCACCACCACCACCACCACACCGGTAAAACCGGACACATTGAT | 2-tHMGr-T_HMGr_ |
| 2-tHMGr-T-R | ATTTTTCTTTTTTTTAGTTATGACAATTACAACAACAGAAT |  |
| 2-TCYC1-F | GTAATTGTCATAACTAAAAAAAAGAAAAATTTGAAATATAA | 2-T_CYC1_ |
| 2-TCYC1-R | CATAGAATGCTATAAACAGGCCCCTTTTCCTTTGTCGAT |  |
| 2-IDI1-F | AGGAAAAGGGGCCTGTTTATAGCATTCTATGAATTTGCCTG | 2-IDI1 |
| 2-IDI1-R | ACACTATATCAATAATGACTGCCGACAACAATAGTATGC |  |
| 2-PCCW12-F | GTTGTCGGCAGTCATTATTGATATAGTGTTTAAGCGAATGA | 2-P_CCW12_ |
| 2-PCCW12-R | TAAAAATAAAAACCAACTAATACATGAAGAAAAAAAAGCAGACAAAAACATTTTATGGACCTGATGCAATCTATTGGCGTCTGATTTCCGTTTTG |  |
| 3-PCCW12-F | TCACTGCTGGTCCTTGCCGACCAGCGTATACAATCTCGATAGTTGGTTTCCCGTTCTTTCCACTCCCGTCCTATTGGCGTCTGATTTCCGTTTTG | 3-P_CCW12_ |
| 3-PCCW12-R | CTTTTTCTGAAGCCATTATTGATATAGTGTTTAAGCGAATGA |  |
| 3-ERG20-F | TAAACACTATATCAATAATGGCTTCAGAAAAAGAAATTAGGAG | 3-ERG20 |
| 3-ERG20-R | AGGAAAAGGGGCCTGTCTATTTGCTTCTCTTGTAAACTTTG |  |
| 3-TCYC1-F | CAAGAGAAGCAAATAGACAGGCCCCTTTTCCTTTGTCGAT | 3-T_CYC1_ |
| 3-TCYC1-R | TTGGGGATATTGGCTAAAAAAAAGAAAAATTTGAAATA |  |
| 3-TTDH3-F | AATTTTTCTTTTTTTTAGCCAATATCCCCAAAATTATTAAG | 3-T_TDH3_ |
| 3-TTDH3-R | CTCTATTGTCATTGAAAAGATATGAGTGAATTTACTTTAAATCTTGCAT |  |
| 3-ERG10-F | TAAAGTAAATTCACTCATATCTTTTCAATGACAATAGAG | 3-ERG10 |
| 3-ERG10-R | CACATAAACAAACAAAATGTCTCAGAACGTTTACATTGTATC |  |
| 3-PTDH3-F | TAAACGTTCTGAGACATTTTGTTTGTTTATGTGTGTTTATTCG | 3-P_TDH3_ |
| 3-PTDH3-R | TAGATATATACTCAGTATTCGTTTTTATAACGTTCGCTGCACTGGGGGCCAAGCACAGGGCAAGATGCTTTCATTATCAATACTGCCATTTCA |  |
| 4-PTDH3-F | TAAGCAAAAAATTGAAAGTTTACTAACCTTTCATTAAAGAGAAATAACAATATTATAAAAAGCGCTTAAATCATTATCAATACTGCCATTTCAA | 4-P_TDH3_ |
| 4-PTDH3-R | TAGTTGAGAGTTTCATTTTGTTTGTTTATGTGTGTTTATTCGA |  |
| 4-ERG13-F | CACATAAACAAACAAAATGAAACTCTCAACTAAACTTTGT | 4-ERG13 |
| 4-ERG13-R | GATTTAAAGTAAATTCACTTATTTTTTAACATCGTAAGATCTTC |  |
| 4-TTDH3-F | CGATGTTAAAAAATAAGTGAATTTACTTTAAATCTTGCAT | 4-T_TDH3_ |
| 4-TTDH3-R | ACGCGTGGCTTTTTTTTAGCCAATATCCCCAAAATTATTAAG |  |
| 4-TB-F | TTGGGGATATTGGCTAAAAAAAAGCCACGCGTGTGCACCT | 4-T_B_ |
| 4-TB-R | CAAGAGAAGCAAATAGCAGTGCTTTTAACTAAGAATTATTAG |  |
| 4-ERG20-F | TAGTTAAAAGCACTGCTATTTGCTTCTCTTGTAAACTTTG | 4-ERG20 |
| 4-ERG20-R | CAACAAATATAAAACAATGGCTTCAGAAAAAGAAATTAGGAG |  |
| 4-PPGK1-F | CTTTTTCTGAAGCCATTGTTTTATATTTGTTGTAAAAAGTAG | 4-P_PGK1_ |
| 4-PPGK1-R | TATCTGGAGGTCCTGTGTTCGATCCACAGAATTCGCATATTTTTTTAACGATTTAAAATCATTAGTTTATGTTTGCAAAAAGAACAAAACTGAA |  |
| 5-PTDH3-F | ATAATTTTACTTAATAGCTTTTCATAAATAATAGAATCACAAACAAAATTTACATCTGAGTTAAACAATCTCATTATCAATACTGCCATTTCAAAG | 5-P_TDH3_ |
| 5-PTDH3-F | CTTGAGTTCACCCATTTTGTTTGTTTATGTGTGTTTATTCG |  |
| 5-Mae1-F | CACATAAACAAACAAAATGGGTGAACTCAAGGAAATCTTGA | 5-Mae1 |
| 5-Mae1-R | TAAAGTAAATTCACTTAAACGCTTTCATGTTCACTACTAG |  |
| 5-TTDH3-F | CATGAAAGCGTTTAAGTGAATTTACTTTAAATCTTGCATT | 5-T_TDH3_ |
| 5-TTDH3-R | TTTATCATTATTGACTAGCACATCGAATCTTAAAATACACTATTATTCAGCACTACGGTTTAGCGTGAAAAGCCAATATCCCCAAAATTATTAAG |  |
| 6-PTEF1-F | GTTCAATAAAGGGCACGTTATCAATTGTTAAAGGCAAAGAATCAGAATTAAATCATAGCAAACGACCAAACATAGCTTCAAAATGTTTCTACTCCT | 6-P_TEF1_ |
| 6-PTEF1-R | ACCAGTAGTGATCATCTTAGATTAGATTGCTATGCTTTCT |  |
| 6-ACCS-F | GCAATCTAATCTAAGATGATCACTACTGGTACTCACCACG | 6-ACCS |
| 6-ACCS-R | CTTAGTTAAAAGCACTGTTATCTAGTCAACAAAGCCAAACC |  |
| 6-TB-F | CTTTGTTGACTAGATAACAGTGCTTTTAACTAAGAATTATTAG | 6-T_B_ |
| 6-TB-R | TGTTATTCCATACAGAACAATAAATACGTATATTTCGTATGTCATGTGGAGTATATATTCTTTTTTATTCAAAAAAAAGCCACGCGTGTGCACCT |  |
| 7-PTDH3-F | AAAGTCTTGACTAAATAAACAATTTGTTAATATCCTAATTCGGTAAAGCTTTGTCGAGACATTAACAAAATCATTATCAATACTGCCATTTCAAAG | 7-P_TDH3_ |
| 7-PTDH3-R | CAAGTTAGCGTTCATTTTGTTTGTTTATGTGTGTTTATTCG |  |
| 7-RpMatB-F | CACATAAACAAACAAAATGAACGCTAACTTGTTTGCTAGGT | 7-RpMatB |
| 7-RpMatB-R | CAAAGGAAAAGGGGCCTGTTCATTTATAAATATCCTTGTAAGTC |  |
| 7-TCYC1-F | GGATATTTATAAATGAACAGGCCCCTTTTCCTTTGTCGATA | 7-T_CYC1_ |
| 7-TCYC1-R | AAAGAAAACGAACTAACTAATGTTTAAGTAAAAGAACAAAAAGGTAGACCAATGTAGCGCTCTTACTTTAAAAAAAAAGAAAAATTTGAAATATA |  |
| 8-PTDH3-F | TTTCTATTACAACTATTAGCTCTAAATCCATATCCTCATAAGCAGCAATCAATTCTATCTATACTTTAAAAAGACCCGTTGAAAAGAACTTACC | 8-P_TDH3_ |
| 8-PTDH3-R | GGTGGTGGTGCATTTTGTTTGTTTATGTGTGTTTATTCG |  |
| 8-LsGAS-F | CATAAACAAACAAAATGCACCACCACCACCACCACGCTGCTGTTGACACCAACGCTA | 8-LsGAS |
| 8-LsGAS-R | TGGTTTTTTTCATCCAAAATATTAAATTTTACTTTTATTACATACAACTTTTTAAACTAATATACACATTTTACATAGAAACAGAACCAACGAAC |  |
| HO-gRNA-F | GATCGTAAGGCTTCATTATGGAGA |  |
| HO-gRNA-R | AAACTCTCCATAATGAAGCCTTAC |  |
| Ygr250c-gRNA-F | GATCGTTAAGGTCATTACTGTTGG |  |
| Ygr250c-gRNA-R | AAACCCAACAGTAATGACCTTAAC |  |
| Gal80-gRNA-F | GATCGCATCATACCCCGGGTCTAA |  |
| Gal80-gRNA-R | AAACTTAGACCCGGGGTATGATGC |  |
| Ndt80-gRNA-F | GATCGGGGTCAAGTTGATTGCAAG |  |
| Ndt80-gRNA-R | AAACCTTGCAATCAACTTGACCCC |  |
| QHS-gRNA-F | GATCGCGGCTGTTTCAGAGCTTCC |  |
| QHS-gRNA-R | AAACGGAAGCTCTGAAACAGCCGC |  |
| P1-PTDH3-F | gtaccgggccccccctcgaggtcgacAAGACCCGTTGAAAAGAACTTACCTG | P1-T_TDH3_ |
| P1-PTDH3-R | CATAAGAAATTCGGaagcttTTTGTTTGTTTATGTGTGTTTATTC |  |
| P1-TADH1-F | CACACATAAACAAACAAAaagcttCCGAATTTCTTATGATTTATGATT | P1-T_ADH1_ |
| P1-TADH1-R | gctctagaactagtggatccgatatcCCGGTAGAGGTGTGGTCAATAAGAGC |  |
| P1-RlMatC-F | AACACACATAAACAAACAAAaagcttATGGGCATTGAAATTTTAGCAATTG | P1-RlMatC |
| P1-RlMatC-R | ATAAATCATAAGAAATTCGGaagcttTTACACTAAGCCCGGAACAACAAAC |  |
| P2-Mae1-F | AACACACATAAACAAACAAAaagcttatgggtgaactcaaggaaatcttga | P2-Mae1 |
| P2-Mae1-R | ATAAATCATAAGAAATTCGGaagcttTTAAACGCTTTCATGTTCACTACTAG |  |
| P4-MdcF-F | AACACACATAAACAAACAAAaagcttATGACCTATGTGATTATTCATGCACT | P4-MdcF |
| P4-MdcF-R | ATAAATCATAAGAAATTCGGaagcttTTACAGGCCGCTGGTTAAAGAAATA |  |
| P5-MadLM-F | AACACACATAAACAAACAAAaagcttATGATTATTTATGGTGTGGGTCTGT | P5-MadLM |
| P5-MadLM-R | ATAAATCATAAGAAATTCGGaagcttTTATGCGCCAATCAGGGCTTTAACT |  |
| P6-*dctPQM*-F | AACACACATAAACAAACAAAaagcttATGAGCAGCTTTCGTCGCAAACTGA | P6-*dctPQM* |
| P6-*dctPQM*-R | ATAAATCATAAGAAATTCGGaagcttTTACTGCACGGTACCCAGCATCTGA |  |
| P7-*matPQM*-F | AACACACATAAACAAACAAAaagcttATGCTGACCCGTCGTATTCTGGGT | P7-*matPQM* |
| P7-*matPQM*-R | ATAAATCATAAGAAATTCGGaagcttTTACATGCCCAGCAGATTCGGCAGT |  |
| P8-TTDH3-F | tatagggcgaattgggtaccAGCCAATATCCCCAAAATTATTAAG | P8-T_TDH3_ |
| P8-TTDH3-R | CACCACCACCACTAAGTGAATTTACTTTAAATCTTGCATT |  |
| P8-Mae1-F | TAAAGTAAATTCACTTAGTGGTGGTGGTGGTGGTGAACGCTTTCATGTTCACTACTAGGA | P8-Mae1 |
| P8-Mae1-R | CACATAAACAAACAAAATGGGTGAACTCAAGGAAATCTTGA |  |
| P8-PTDH3-F | CCTTGAGTTCACCCATTTTGTTTGTTTATGTGTGTTTATTC | P8-P_TDH3_ |
| P8-PTDH3-R | ctcgagggggggcccggtaccTCATTATCAATACTGCCATTTCA |  |
| P8-PTEF1-F | ggtaccgggccccccctcgaggtcgacCATAGCTTCAAAATGTTTCTACTCCT | P8-P_TEF1_ |
| P8-PTEF1-R | GAACAGGTTGGCGTTCATCTTAGATTAGATTGCTATGCTTTCT |  |
| P8-AtMatB-F | CACATAAACAAACAAAATGACTGCTACTACTACTTTGAAGTC | P8-AtMatB |
| P8-AtMatB-R | TAAAGTAAATTCACTCAGTGGTGGTGGTGGTGGTGCTCTTGGTTCTCCAGGGACTTCT |  |
| P8-TADH1-F | GACATCTACAAGCACCACCACCACCACCACTAACCGAATTTCTTATGATTTATGATT | P8-T_ADH1_ |
| P8-TADH1-R | gatatcaagcttatcgataccgtcgacCCGGTAGAGGTGTGGTCAATAAGAGC |  |
| P9-TTDH3-F | tatagggcgaattgggtaccAGCCAATATCCCCAAAATTATTAAG | P9-T_TDH3_ |
| P9-TTDH3-R | CACCACCACCACTAAGTGAATTTACTTTAAATCTTGCATT |  |
| P9-Mae1-F | TAAAGTAAATTCACTTAGTGGTGGTGGTGGTGGTGAACGCTTTCATGTTCACTACTAGGA | P9-Mae1 |
| P9-Mae1-R | CACATAAACAAACAAAATGGGTGAACTCAAGGAAATCTTGA |  |
| P9-PTDH3-F | CCTTGAGTTCACCCATTTTGTTTGTTTATGTGTGTTTATTC | P9-P_TDH3_ |
| P9-PTDH3-R | ctcgagggggggcccggtaccTCATTATCAATACTGCCATTTCA |  |
| P9-PTEF1-F | ggtaccgggccccccctcgaggtcgacCATAGCTTCAAAATGTTTCTACTCCT | P9-P_TEF1_ |
| P9-PTEF1-R | GAACAGGTTGGCGTTCATCTTAGATTAGATTGCTATGCTTTCT |  |
| P9-BjMatB-F | CACATAAACAAACAAAATGAATCAAGCTGCTAATGCTAAC | P9-BjMatB |
| P9-BjMatB-R | TAAAGTAAATTCACTCAGTGGTGGTGGTGGTGGTGCTCTTGGTTCTCCAGGGACTTCT |  |
| P9-TADH1-F | GACATCTACAAGCACCACCACCACCACCACTAACCGAATTTCTTATGATTTATGATT | P9-T_ADH1_ |
| P9-TADH1-R | gatatcaagcttatcgataccgtcgacCCGGTAGAGGTGTGGTCAATAAGAGC |  |
| P10-TTDH3-F | tatagggcgaattgggtaccAGCCAATATCCCCAAAATTATTAAG | P10-T_TDH3_ |
| P10-TTDH3-R | CACCACCACCACTAAGTGAATTTACTTTAAATCTTGCATT |  |
| P10-Mae1-F | TAAAGTAAATTCACTTAGTGGTGGTGGTGGTGGTGAACGCTTTCATGTTCACTACTAGGA | P10-Mae1 |
| P10-Mae1-R | CACATAAACAAACAAAATGGGTGAACTCAAGGAAATCTTGA |  |
| P10-PTDH3-F | CCTTGAGTTCACCCATTTTGTTTGTTTATGTGTGTTTATTC | P10-P_TDH3_ |
| P10-PTDH3-R | ctcgagggggggcccggtaccTCATTATCAATACTGCCATTTCA |  |
| P10-PTEF1-F | ggtaccgggccccccctcgaggtcgacCATAGCTTCAAAATGTTTCTACTCCT | P10-P_TEF1_ |
| P10-PTEF1-R | ACAAGTTAGCGTTCATCTTAGATTAGATTGCTATGCTTTCT |  |
| P10-RpMatB-F | AGCAATCTAATCTAAGATGAACGCTAACTTGTTTGCTAGGT | P10-RpMatB |
| P10-RpMatB-R | ACAAGTTAGCGTTCATCTTAGATTAGATTGCTATGCTTTCT |  |
| P10-TADH1-F | CAAGGATATTTATAAATCCAAGCTGTGACCGAATTTCTTATGATTTATGAT | P10-T_ADH1_ |
| P10-TADH1-R | gatatcaagcttatcgataccgtcgacCCGGTAGAGGTGTGGTCAATAAGAGC |  |
| P11-TB-F | gtcgacggtatcgataagcttgatatcAAAAAAAAGCCACGCGTGTGCACCT | P11-T_B_ |
| P11-TB-R | GCGCCGACGGCGCACCACCACCACCACCACTGACAGTGCTTTTAACTAAGAATTATTAG |  |
| P11-MatA-F | AGTTAAAAGCACTGTCAGTGGTGGTGGTGGTGGTGCGCCGTCGGCGCGGCCTGCGGGGC | P11-MatA |
| P11-MatA-R | CAACAAATATAAAACAATGAAGCTGATGCCGATAGCCACCA |  |
| P11-PPGK1-F | TCGGCATCAGCTTCATTGTTTTATATTTGTTGTAAAAAGT | P11-P_PGK1_ |
| P11-PPGK1-R | atcccccgggctgcaggaattcgatatcGTTTGCAAAAAGAACAAAACTGA |  |
| P12-TB-F | gtcgacggtatcgataagcttgatatcAAAAAAAAGCCACGCGTGTGCACCT | P12-T_B_ |
| P12-TB-R | TTGTTGACTAGACACCACCACCACCACCACTAACAGTGCTTTTAACTAAGAATTATTAG |  |
| P12-ACCS-F | TAGTTAAAAGCACTGTTAGTGGTGGTGGTGGTGGTGTCTAGTCAACAAAGCCAAACCCA | P12-ACCS |
| P12-ACCS-R | CAACAAATATAAAACAATGATCACTACTGGTACTCACCACG |  |
| P12-PPGK1-F | TACCAGTAGTGATCATTGTTTTATATTTGTTGTAAAAAGT | P12-P_PGK1_ |
| P12-PPGK1-R | atcccccgggctgcaggaattcgatatcGTTTGCAAAAAGAACAAAACTGA |  |
| P13-TTDH3-F | tatagggcgaattgggtaccAGCCAATATCCCCAAAATTATTAAG | P13-T_TDH3_ |
| P13-TTDH3-R | CACCACCACCACTAAGTGAATTTACTTTAAATCTTGCATT |  |
| P13-Mae1-F | TAAAGTAAATTCACTTAGTGGTGGTGGTGGTGGTGAACGCTTTCATGTTCACTACTAGGA | P13-Mae1 |
| P13-Mae1-R | CACATAAACAAACAAAATGGGTGAACTCAAGGAAATCTTGA |  |
| P13-PTDH3-F | CCTTGAGTTCACCCATTTTGTTTGTTTATGTGTGTTTATTC | P13-P_TDH3_ |
| P13-PTDH3-R | ctcgagggggggcccggtaccTCATTATCAATACTGCCATTTCA |  |
| P13-PTEF1-F | ggtaccgggccccccctcgaggtcgacCATAGCTTCAAAATGTTTCTACTCCT | P13-P_TEF1_ |
| P13-PTEF1-R | ACAAGTTAGCGTTCATCTTAGATTAGATTGCTATGCTTTCT |  |
| P13-RpMatB-F | AGCAATCTAATCTAAGATGAACGCTAACTTGTTTGCTAGGT | P13-RpMatB |
| P13-RpMatB-R | ACAAGTTAGCGTTCATCTTAGATTAGATTGCTATGCTTTCT |  |
| P13-TADH1-F | CAAGGATATTTATAAATCCAAGCTGTGACCGAATTTCTTATGATTTATGAT | P13-T_ADH1_ |
| P13-TADH1-R | gatatcaagcttatcgataccgtcgacCCGGTAGAGGTGTGGTCAATAAGAGC |  |
| P13-TB-F | gtcgacggtatcgataagcttgatatcAAAAAAAAGCCACGCGTGTGCACCT | P13-T_B_ |
| P13-TB-R | GCGCCGACGGCGCACCACCACCACCACCACTGACAGTGCTTTTAACTAAGAATTATTAG |  |
| P13-MatA-F | AGTTAAAAGCACTGTCAGTGGTGGTGGTGGTGGTGCGCCGTCGGCGCGGCCTGCGGGGC | P13-MatA |
| P13-MatA-R | CAACAAATATAAAACAATGAAGCTGATGCCGATAGCCACCA |  |
| P13-PPGK1-F | TCGGCATCAGCTTCATTGTTTTATATTTGTTGTAAAAAGT | P13-P_PGK1_ |
| P13-PPGK1-R | atcccccgggctgcaggaattcgatatcGTTTGCAAAAAGAACAAAACTGA |  |
| P14-TTDH3-F | tatagggcgaattgggtaccAGCCAATATCCCCAAAATTATTAAG | P14-T_TDH3_ |
| P14-TTDH3-R | CACCACCACCACTAAGTGAATTTACTTTAAATCTTGCATT |  |
| P14-Mae1-F | TAAAGTAAATTCACTTAGTGGTGGTGGTGGTGGTGAACGCTTTCATGTTCACTACTAGGA | P14-Mae1 |
| P14-Mae1-R | CACATAAACAAACAAAATGGGTGAACTCAAGGAAATCTTGA |  |
| P14-PTDH3-F | CCTTGAGTTCACCCATTTTGTTTGTTTATGTGTGTTTATTC | P14-P_TDH3_ |
| P14-PTDH3-R | ctcgagggggggcccggtaccTCATTATCAATACTGCCATTTCA |  |
| P14-PTEF1-F | ggtaccgggccccccctcgaggtcgacCATAGCTTCAAAATGTTTCTACTCCT | P14-P_TEF1_ |
| P14-PTEF1-R | ACAAGTTAGCGTTCATCTTAGATTAGATTGCTATGCTTTCT |  |
| P14-RpMatB-F | AGCAATCTAATCTAAGATGAACGCTAACTTGTTTGCTAGGT | P14-RpMatB |
| P14-RpMatB-R | ACAAGTTAGCGTTCATCTTAGATTAGATTGCTATGCTTTCT |  |
| P14-TADH1-F | CAAGGATATTTATAAATCCAAGCTGTGACCGAATTTCTTATGATTTATGAT | P14-T_ADH1_ |
| P14-TADH1-R | gatatcaagcttatcgataccgtcgacCCGGTAGAGGTGTGGTCAATAAGAGC |  |
| P14-TB-F | gtcgacggtatcgataagcttgatatcAAAAAAAAGCCACGCGTGTGCACCT | P14-T_B_ |
| P14-TB-R | TTGTTGACTAGACACCACCACCACCACCACTAACAGTGCTTTTAACTAAGAATTATTAG |  |
| P14-ACCS-F | TAGTTAAAAGCACTGTTAGTGGTGGTGGTGGTGGTGTCTAGTCAACAAAGCCAAACCCA | P14-ACCS |
| P14-ACCS-R | CAACAAATATAAAACAATGATCACTACTGGTACTCACCACG |  |
| P14-PPGK1-F | TACCAGTAGTGATCATTGTTTTATATTTGTTGTAAAAAGT | P14-P_PGK1_ |
| P14-PPGK1-R | atcccccgggctgcaggaattcgatatcGTTTGCAAAAAGAACAAAACTGA |  |

**Table S3** All primers used in transcription analysis of genes.

| **Primers** | **Sequence(5’→3’)** |
| --- | --- |
| QHS-F | CTTCTGGGCTCTGGGTGTTT |
| QHS-R | TCCAGAACGGTAGCCAGAGA |
| Mae1-F | TCCGTGCGTTTCTCGTCAATGATC |
| Mae1-R | TTCAGGTGGGAAGGTAGGGTTAAGG |
| RpMatB-F | TGGTCTGCTAAAACTGGTCATGCTG |
| RpMatB-R | TGGAACCCTGTCACCGTCGTAG |
| AtMatB-F | TGATCAACCACAGGCTGAAC |
| AtMatB-R | AAGAACCAGACTGAGAAGAG |
| BjMatB-F | GCTGCTAATGCTAACTTGTT |
| BjMatB-R | GGCAATCAAATCACCATAAG |
| ACCS-F | CTTGGCTGTTGCTGCTGGTAGAG |
| ACCS-R | TGTGGAGCGTCTGGAGTAGAAGTAG |
| ERG10-F | GCTGTTGCTCTAGGTCACCCATTG |
| ERG10-R | GGAAGCACCACCACCACCATTAC |
| ERG13-F | CGGTACTGTTGCTATGTGGATCGG |
| ERG13-R | TCGTAGGCGTGTTCCATGTAAGAAG |
| tHMGr-F | GTAGCAGAAGCGGCGGGTTTAG |
| tHMGr-R | CCGTTGGCTAATGACAGAGGAAGAG |
| ERG20-F | AGGACTCAGTCGCAGAAGCC |
| ERG20-R | GCCACGAGACTCATCGACCT |
| HXT7-F | TCGGTAAGATCGAAGAAGCCAAACG |
| HXT7-R | TCTCTGCCTCTACACCAGCCAAG |
| FBP1-F | TCAGACTGCTACCAGACTCATCAGG |
| FBP1-R | AACCCATCAACTCCATCACCCAATG |
| HXK1-F | TGTTTGTGGTATTGCCGCTATTTGC |
| HXK1-R | CTTGCGTCACCAGTCCATCCATAG |
| GLK1-F | GGGATGTTCTTGGGTGAGGTGTTG |
| GLK1-R | AGTCAAGTGGCGAGGAAGTTGTTC |
| PDC1-F | TGCTTACGCCGCTGATGGTTAC |
| PDC1-R | CAGACAATTCACCGACACCGAAGG |
| ICL1-F | AACTTCTCTCGTGACTTTGCCAAGG |
| ICL1-R | ACATCAACACCATCGTCCATTTCCC |
| MLS1-F | AGAGCTGACAAATGGACATGATGGG |
| MLS1-R | TTCCAGCAGATTAGCAGCCGTTAC |
| MCT1-F | ATTCGGAGAAATTGTCCCCAAGTGG |
| MCT1-R | CCTCTGCCGCCTTGTGATAAGC |
| ACC1-F | CACGGTGGTCACACGGTCATATC |
| ACC1-R | ATGGCGACGAATTGGACGGTTC |
| ADH2-F | GGGTAACGAATCCAACTGTCCTCAC |
| ADH2-R | CAGCGTCAGCGGTAGCGTATTC |
| ACS1-F | GCCTCATTCCCCTTCTTCGGTATTG |
| ACS1-R | CCTCTGCGTGGCTGGTGTTAAG |
| LsGAS-F | GCGTTACTCTCTGGCTCGTATCATC |
| LsGAS-R | GTAGCGTAAGCGTCGTAGGTGTC |
