# Supplementary material for: Switching carbon metabolic flux for enhancing the production of sesquiterpene-based high-density biofuel precursor in Saccharomyces cerevisiae
Source: Biotechnol Biofuels Bioprod. 2023 Aug 4;16:124. doi: 10.1186/s13068-023-02370-8 (PMC10403917; doi:10.1186/s13068-023-02370-8)
Supplement: Supplementary file 2 — Additional file 2: Figure S1. Malonate transport efficiency of strain Mae I with different concentrations of malonate. Figure S2. Detection of β-caryophyllene by GC–MS in 13C isotopic tracer assay. (A) The total ion chromatogram of β-caryophyllene. (B) The mass spectrum of the β-caryophyllene peak in (A). Figure S3. Time courses of cell growth (A) and β-elemene production (B) during shake-flask fermentation in culture medium under different fermentation conditions. Error bars indicate the standard deviations of three biological replicates. [file 13068_2023_2370_MOESM2_ESM.docx]

**Switching carbon metabolic flux for enhanced production of sesquiterpene-based high-density biofuel precursor in engineered yeast**

Bo Liang^a,b^, Qun Yang^a,b^, Xinping Zhang^a,b^, Yukun Zhao^c^, Yunhui Liu^a,b^, Jianming Yang^a,b^*, Zhaobao Wang^a,b^*

^a^Energy-rich Compounds Production by Photosynthetic Carbon Fixation Research Center, Qingdao Agricultural University, Qingdao, China.

^b^Shandong Key Lab of Applied Mycology, College of Life Sciences, Qingdao Agricultural University, Qingdao, China

^c^Pony Testing International Group, Qingdao, China

^*^Correspondence: [yjming888@126.com](mailto:yjming888@126.com), [wangzhaobao123@126.com](mailto:wangzhaobao123@126.com)

Bo Liang, Qun Yang and Xinping Zhang contributed equally to this work


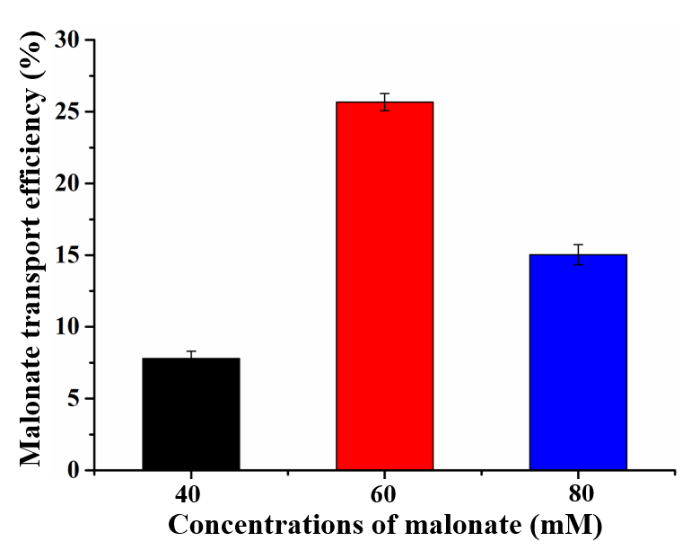


**Fig. S1.** Malonate transport efficiency of strain Mae I with different concentrations of malonate.


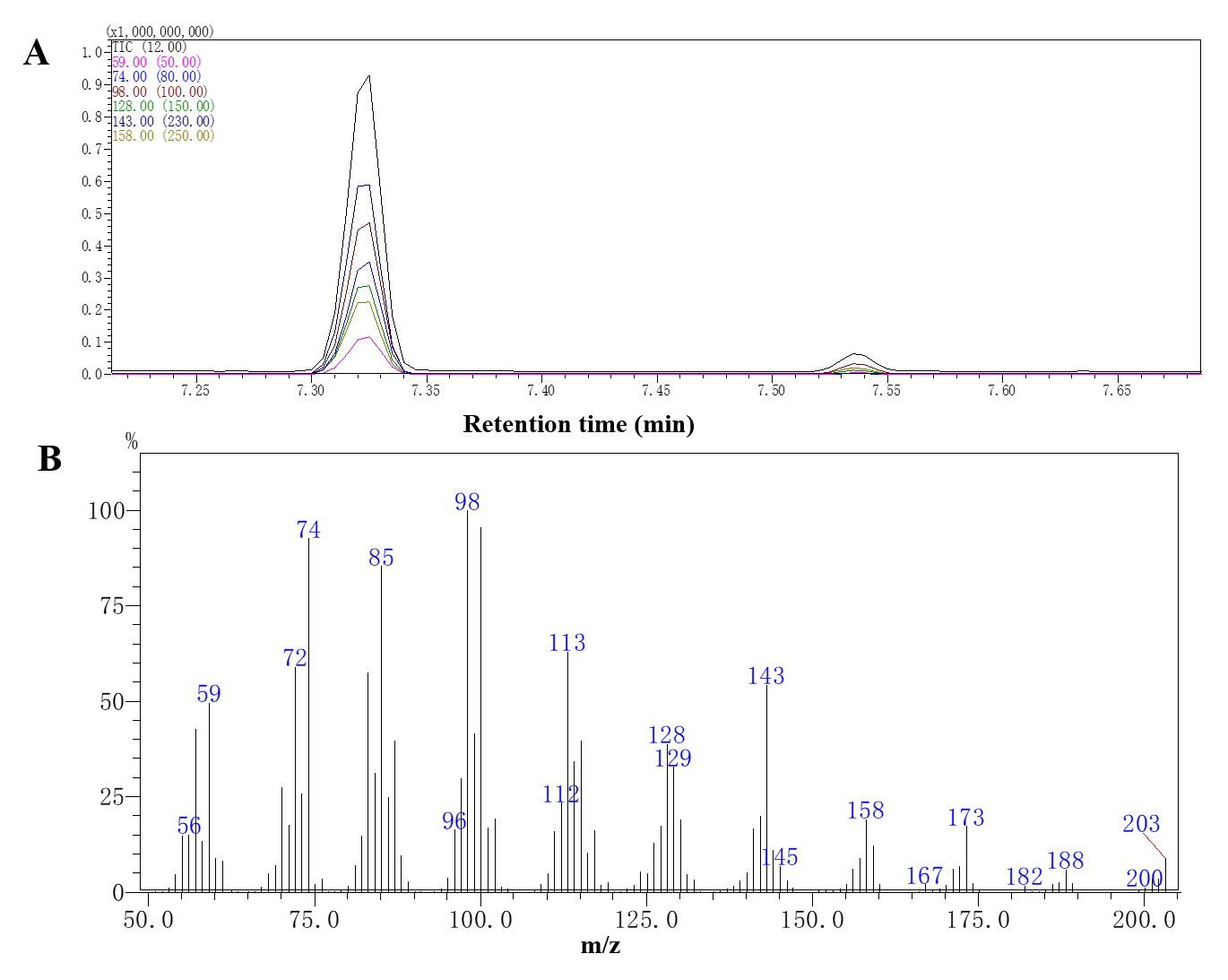


**Fig. S2.** Detection of β-caryophyllene by GC-MS in ^13^C isotopic tracer assay. (A) The total ion chromatogram of β-caryophyllene. (B) The mass spectrum of the β-caryophyllene peak in (A).


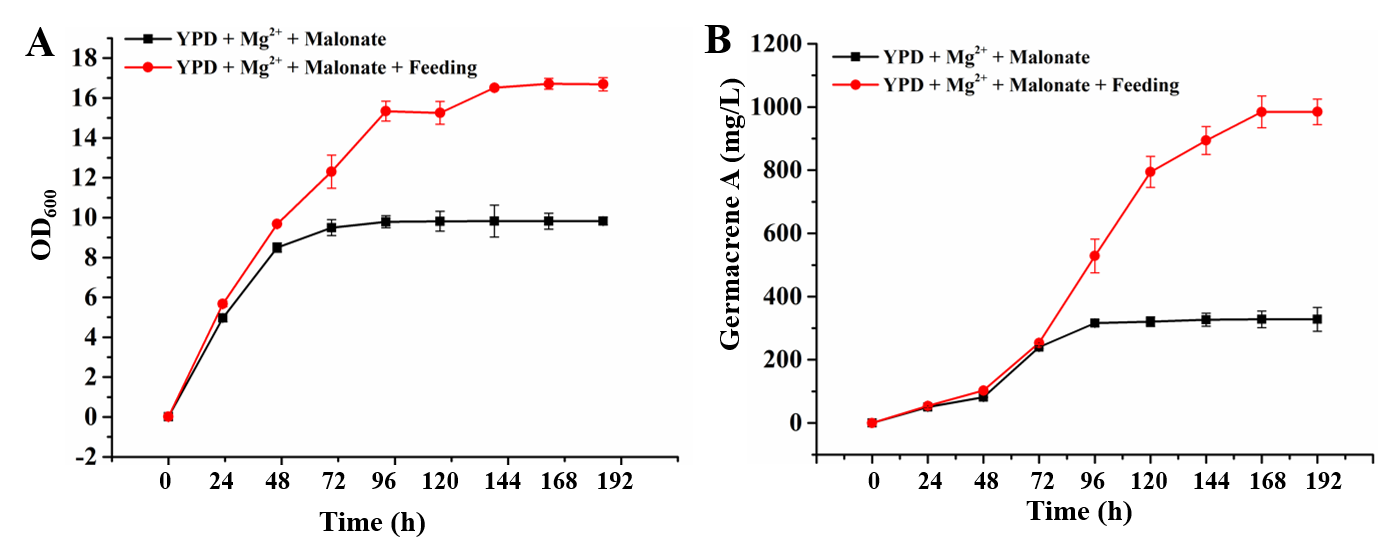


**Fig. S3.** Time courses of cell growth (A) and β-elemene production (B) during shake-flask fermentation in culture medium under different fermentation conditions. Error bars indicate the standard deviations of three biological replicates.
